# Systematic review: Association between circulating microRNA expression & stroke

**DOI:** 10.1177/0271678X221085090

**Published:** 2022-03-03

**Authors:** Josie L Fullerton, Josephine M Thomas, Laura Gonzalez-Trueba, Cara Trivett, Josie C van Kralingen, Stuart M Allan, Terence J Quinn, Lorraine M Work

**Affiliations:** 1 Institute of Cardiovascular & Medical Science, College of Medical, Veterinary and Life Sciences, University of Glasgow, Glasgow, UK; 2 Division of Neuroscience & Experimental Psychology, School of Biological Sciences, Faculty of Biology, Medicine & Health, University of Manchester, UK; 3 Geoffrey Jefferson Brain Research Centre, Manchester Academic Health Science Centre, Northern Care Alliance NHS Group, & University of Manchester, Manchester, UK

**Keywords:** Biomarkers, haemorrhagic, ischaemic, microRNAs, stroke

## Abstract

This systematic review aimed to establish the range and quality of clinical and preclinical evidence supporting the association of individual microRNAs, and the use of microRNA expression in the diagnosis and prognosis of ischaemic or haemorrhagic stroke. Electronic databases were searched from 1993 to October 2021, using key words relevant to concepts of stroke and microRNA. Studies that met specific inclusion and exclusion criteria were selected for data extraction. To minimise erroneous associations, findings were restricted to microRNAs reported to change in more than two independent studies. Of the papers assessed, 155 papers reported a change in microRNA expression observed in more than two independent studies. In ischaemic studies, two microRNAs were consistently differentially expressed in clinical samples (miR-29b & miR-146a) and four were altered in preclinical samples (miR-137, miR-146a, miR-181b & miR-223-3p). Across clinical and preclinical haemorrhagic studies, four microRNAs were downregulated consistently (miR-26a, miR-126, miR-146a & miR-155). Across included studies, miR-126 and miR-146a were the only two microRNAs to be differentially expressed in clinical and preclinical cohorts following ischaemic or haemorrhagic stroke. Further studies, employing larger populations with consistent methodologies, are required to validate the true clinical value of circulating microRNAs as biomarkers of ischaemic and haemorrhagic stroke.

## Introduction

At present, the diagnosis of ischaemic and haemorrhagic stroke primarily relies upon clinical assessment, supplemented by neuroimaging. Within the first hours of suspected stroke, initial assessment may not be performed by a stroke specialist and even for the specialist, hyperacute clinical assessment is challenging. On initial presentation 2-26% of strokes are misdiagnosed resulting in delayed medical intervention, with devastating health and financial consequences for patients and their families.^1^

In suspected stroke, accurate, and timely diagnosis is vital, as current treatment options are time sensitive. If delayed, impeded stroke diagnosis is associated with adverse clinical outcome, increased risk of stroke recurrence, and increased mortality rates.^2^ The development of a non-invasive method of rapidly testing patients with a suspected stroke would be invaluable, accelerating stroke diagnosis and implementation of appropriate medical intervention; thus, improving patient outcomes. At present, there are no predictors or biomarkers of ischaemic or haemorrhagic stroke that are clinically routine; therefore, it is essential to develop a low-cost, sensitive, and reliable method of stroke diagnosis, but also improve current therapy options for both stroke subtypes.^3^

In the setting of stroke, microRNAs hold great potential to act as a novel diagnostic, prognostic, and therapeutic tool.^4^ MicroRNAs are small, non-coding, single-stranded RNAs that play a vital role in health and disease.^5^ At the post-transcriptional level, microRNAs regulate gene expression by regulating target messenger RNA (mRNA) resulting in altered levels of target protein(s). Single or several microRNAs can bind to the 3′ untranslated regions (UTRs) of target mRNA leading to it’s degradation or repression or inhibition of translation/transcription.^6^ A single microRNA holds the potential to regulate thousands of downstream target genes, influencing entire gene networks and protein synthesis.^7^ These endogenous small molecules are presents in all types of body fluid including serum, plasma, urine, and cerebrospinal fluid (CSF).^8^

Emerging data from preclinical studies suggests an association between circulating levels of certain microRNA and stroke,^9^ similar associations have been seen clinically.^10^ Evidence shows that circulating microRNAs play a key role in the neuropathological processes triggered by stroke. Therefore, post-stroke, microRNA levels could be assessed as a clinical biomarker, while also providing insight into underlying pathological mechanisms of stroke.^11^ Furthermore, modulating levels of specific microRNA suggest that this approach may be used as a novel therapeutic intervention for the treatment of stroke.^12^

The primary aim of this systematic review was to establish the current range and quality of both clinical and preclinical evidence supporting the association of individual microRNAs within ischaemic and haemorrhagic stroke. Secondly, we aimed to assess the evidence to support the use of microRNA expression in the diagnosis and prognosis of ischaemic or haemorrhagic stroke.

## Materials and methods

### Study design

This systematic review was performed in line with preferred reporting items for systematic review and meta-analysis (PRISMA) statement, where appropriate. A prespecified protocol was developed following the PRISMA-P statement and was published in the SyRF Systematic Review Facility (http://syrf.org.uk/protocols/: – published 22.8.17).

### Search strategy

A systematic literature search was conducted using Medline (via Ovid MEDLINE 1993–2021), Embase (via Ovid EMBASE 1993–2021), and Web of Science via EBSCO, representing a broad field of clinical and translational cerebrovascular research. For the selected databases, a search syntax was developed and applied (Supplementary information).

### Inclusion and exclusion criteria

The search was limited to original papers published in peer reviewed scientific journals during or after 1993 until October 2021, inclusively; 1993 was set as a cut-off point as microRNAs were first described in this year.^13^ To further validate our results and to avoid false positive associations, findings were restricted to microRNA expression reported to change in more than two independent studies. The studies included in this review were selected based on the specific inclusion and exclusion criteria as prespecified in the protocol (http://syrf.org.uk/protocols/; Supplementary information).

### Paper screening

To identify eligible studies, titles and abstracts obtained from the search strategy were screened by two independent researchers (JLF, LMW) using the SyRF screening application. Following this, eligible studies were then selected for inclusion after full-text analysis by three independent researchers (JLF, JMT, LGT). A consensus was met between reviewers to resolve any inclusion/exclusion differences and all included papers are listed in the Supplementary information.

### Data extraction

Relevant data were extracted onto a standardized, piloted, proforma spreadsheet by three independent researchers (JLF, JMT, LGT; Supplementary information). Additional methodology described online or in paper supplements were assessed, where available. If data were not provided in the article or the supplementary information, the corresponding author was contacted to request the missing data. If microRNA data were not numerically stated, data were extrapolated from published figures using WebPlotDigitizer (https://apps.automeris.io/wpd/).

Extracted data were categorised by ischaemic or haemorrhagic stroke subtype, and by clinical or preclinical approach. Only exact microRNA nomenclature was accepted, differences between 3p and 5p strands were not included. All data from primary screening studies were excluded to avoid repeated data sets. In the event that only a subset of patients or animals met the inclusion criteria, the characteristics of these groups were reported.

### Quality assessment

The quality and validity of the included studies were assessed using the Collaborative Approach to Meta Analysis and Review of Animal Experimental Studies (CAMARADES) checklist. This tool was modified to include stroke-specific evaluations. For clinical and preclinical studies evaluations were made by two independent assessors (JLF, JMT; Supplementary information). Evaluations were answered with “yes”, “no” or “unclear” and were scored as the total number of yes responses. Studies were deemed as high quality, moderate quality, or low quality if the average assessment scores were >75%, 50–75% or <50%, respectively (https://www.ed.ac.uk/clinical-brain-sciences/research/camarades).

## Results

### Literature search

**Figure 1.**
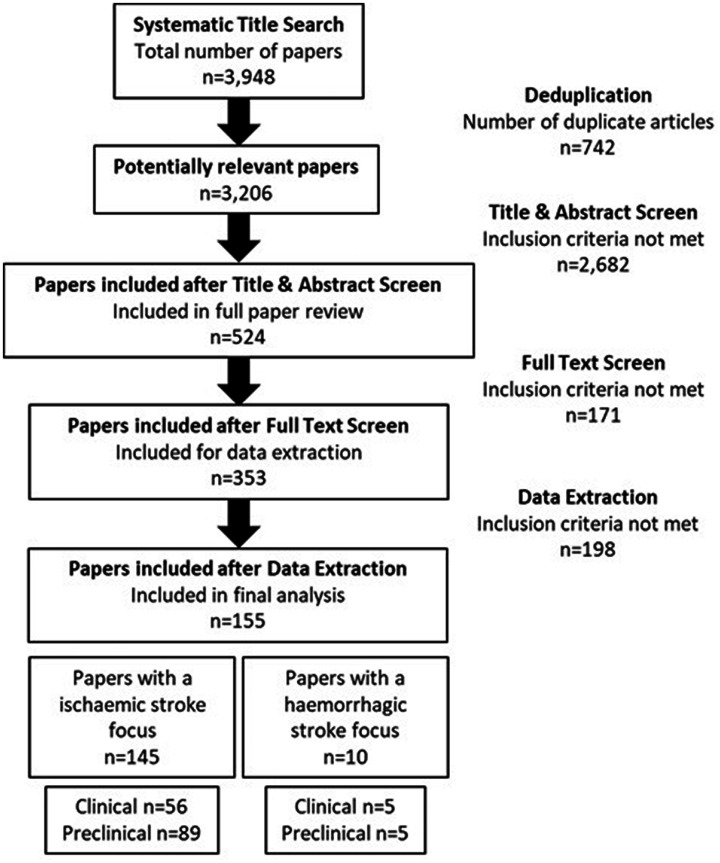
Strategy implemented in the focused literature search and screening process. Papers were selected based on a set of inclusion and exclusion criteria. Following further comprehensive review, papers that lacked a method of qualitative analysis, such as qRT-PCR, were excluded from final analysis; this yielded 353 papers. Of these, 155 reported a change in microRNA expression in more than two independent studies.

The initial database search yielded 3,948 papers for screening. The full texts of 524 studies were assessed, 171 of these were excluded mainly due to the lack of healthy controls or quantitative validation, such as quantitative real-time polymerase chain reaction (qRT-PCR). A total of 353 studies satisfied the inclusion criteria and were taken forward to data extraction (Figure 1).

To account for replication validation of altered microRNA expression, only microRNA(s) which were reported to be altered in more than two independent studies were included for further analysis, a criterion met by 155 papers. Of these studies, 145 had an ischaemic stroke focus (5893 participants) and 10 were purely haemorrhagic stroke studies (508 participants) (Figure 1). Complete reference details for those listed in Tables 1–7 (numbered 1–157) are included in Supplementary information.

### Quality assessment

For ischaemic stroke studies, the quality and validity assessment of the CAMARADES checklist suggested that 11% of studies were of high quality, 57% of studies were of moderate quality, and 33% were of low quality. For haemorrhagic stroke studies, the quality and validity assessment found that 14% of studies were of high quality, 54% of studies were of moderate quality, and 32% were of low quality. Two studies with the highest quality score of 88% satisfied most predefined criteria (Supplementary information). Only one ischaemic and one haemorrhagic study, both of moderate quality, included a sample size calculation.

### Ischaemic stroke – clinical studies

#### Study design and methodology

The study design and method of included clinical ischaemic stroke studies are summarised in Table 1. From the included studies, a summary of patient clinical data from ischaemic stroke cases and healthy controls are presented in Table 2.

**Table 1.** Study design of included clinical ischaemic stroke studies.

Ref	Country	Definition of ischaemic stroke	Definition of healthy control	Sample type	Primary screening	microRNA quantification
^1^	China	Unclear	Normal controls	Blood	n/a	18S rRNA controlqRT-PCR, 2^−ΔΔCT^
^2^	China	Clinical diagnosisMRI & MRA	Control with no previous stroke	Blood-derived EVs	n/a	qRT-PCR, CT
^3^	China	MRI & DWI	No prior history of stroke	Serum	n/a	U6 controlqRT-PCR, 2^−ΔCT^
^4^	China	Clinical diagnosisMRI & MRA	Healthy controls	Plasma Platelets	n/a	cel-miR-39 controlqRT-PCR, 2^−ΔCT^
^5^	China	Clinical &radiographic diagnosis	Matched controls, no stroke	Blood	n/a	U6 controlqRT-PCR, 2^−ΔΔCt^
^6^	China	Clinical diagnosis MRI or CTMRA	Vascular risk factors, no history of stroke	Plasma	Pro- & anti-angiogenic miRNAs	U6 controlqRT-PCR, 2^−ΔΔ CT^
^7^	China	Clinical diagnosis MRI or CT	Vascular risk factors, no history of stroke	Plasma	n/a	U6 controlqRT-PCR, 2^−ΔΔ CT^
^8^	Malaysia	MRI or CT	Healthy volunteers	Whole blood	n/a	qRT-PCR18s rRNA control
^9^	China	Clinical diagnosisMRI & CT	Healthy controls	Plasma	n/a	snRNA U6 controlqRT-PCR, Ct
^10^	China	MRI or CT	Healthy persons	Serum	n/a	5s rRNA controlqRT-PCR, 2^−ΔΔCT^
^11^	China	Clinical diagnosis MRI or CT	Healthy controls	Whole blood	n/a	U6 controlqRT-PCR, CT
^12^	China	Clinical diagnosisMRI	Normal controls	Plasma	n/a	cel-miR-39 controlqRT-PCR, 2^−ΔΔCT^
^13^	Korea	Clinical diagnosisMRI	Vascular risk factors without stroke	Plasma	n/a	qRT-PCR, 2^−Δ CT^miR-16 control
^14^	China	Clinical diagnosisMRI or CT	Non-stroke controls	Serum-derived EVs	n/a	cel-miR-39 controlqRT-PCR, Cq
^15^	USA	Clinical diagnosisMRI	Control subjects with vascular risk factors	Blood	n/a	U75 controlqRT-PCR
^16^	USA	Clinical diagnosisMRI or CT	No history of cerebrovascular disease	Blood	n/a	U75 controlqRT-PCR
^17^	China	Unclear	Healthy volunteers	Serum	n/a	U6 & GAPDH controlqRT-PCR, 2^−ΔΔCq^
^18^	China	Clinical diagnosisMRI & MRA	Healthy controls	Natural killer cells	n/a	qRT-PCR, 2^−ΔΔCT^
^19^	Egypt	Unclear	Healthy volunteers	Serum	n/a	snoRD68 geneqRT-PCR, 2^−ΔΔCT^
^20^	UK	Clinical diagnosisMRI or CT	Non-stroke control	Serum-derived EVs	13 TaqMan OpenArray	cel-miR-39 controlqRT-PCR, ΔΔCt
^21^	China	Clinical diagnosisMRI or CT	Age-matched healthy individuals	Plasma	n/a	qRT-PCR
^22^	China	Clinical diagnosisMRI or CT	Healthy volunteers	Serum	miRCURYTM LNA Array (v.18.0)	qRT-PCR, 2^−ΔΔCT^
^23^	China	Clinical diagnosisMRI	Normal healthy controls	Whole blood	RiboArray mi-DETECTM (2042 probes)	Rnu6b & Gapdh controlqRT-PCR, 2^−ΔΔ CT^
^24^	China	Clinical diagnosis MRI or DWI	Healthy controls	Serum	n/a	U6 controlqRT-PCR, 2^−ΔΔ CT^
^25^	China	Neurological deficit	Healthy volunteers	Serum	n/a	cel-miR-39 controlqRT-PCR, 2^−ΔCT^
^26^	China	Clinical diagnosisMRI	Sex-matched healthy controls	Blood	n/a	U6 controlqRT-PCR, 2^−ΔΔCT^
^27^	China	Clinical diagnosisMRI	Healthy volunteers, no risk factors	Blood & CSF	n/a	18S rRNA controlqRT-PCR, ΔΔCT
^28^	China	MRI or CT	Healthy volunteers	Blood	n/a	U6 & GAPDH controlqRT-PCR, 2^−ΔΔCt^
^29^	China	Clinical diagnosisMRI	Healthy controls	Plasma	n/a	U6 controlqRT-PCR, 2^−ΔCT^
^30^	China	Clinical diagnosis MRI	Healthy controls	Plasma, Platelets &Leukocytes	n/a	cel-miR-39 controlqRT-PCR, 2^−ΔΔ CT^
^31^	Denmark	Clinical diagnosisCT	Healthy controls	Blood & CSF	n/a	RNA (Sp6) &DNA (Sp3) controlqRT-PCR, Ct
^32^	Denmark	Neurological examinationCT scan	Control	CSF	Human Panel I assay (372 miRNA)	qRT-PCR, 2^−ΔΔCT^
^33^	China	MRI	No cardio- or cerebrovascular disease	Serum	n/a	qRT-PCR, 2^−ΔCT^
^34^	Singapore	MRI or CT	Normal control	Blood	MicroRNA Microarray Assay	18S rRNA controlqRT-PCR, 2^−ΔCT^
^35^	Singapore	MRI or CT	Healthy controls	Blood	n/a	GAPDH controlqRT-PCR
^36^	China	Unclear	Healthy volunteers	Plasma	Agilent Human miRNA (2006 miRNAs)	Spike-in controlqRT-PCR, 2^−ΔΔCT^
^37^	Germany	Clinical diagnosisMRI or CT	Healthy control subjects	Blood &Serum-derived EVs	RNA Seq	qRT-PCR, Cq
^38^	China	Clinical diagnosisMRI or CT	Healthy controls	Blood	n/a	U6 controlqRT-PCR, 2^−ΔCt^
^39^	China	Clinical & radiographic diagnosis	Control group	Blood	n/a	U6 controlqRT-PCR, 2^−ΔCt^
^40^	China	Unclear	Healthy volunteers	Blood	n/a	qRT-PCR, 2^−ΔΔCT^
^41^	China	Clinical diagnosisMRI or CT	No cerebrovascular disease	Serum	n/a	U6 snRNA controlqRT-PCR, lg 2^−△Ct^
^42^	China	Clinical diagnosisMRI or CT	Healthy controls no history of stroke	Serum	n/a	snRNA U6 &GAPDH controlqRT-PCR, 2^−ΔΔCT^
^43^	China	Clinical diagnosisMRI	Healthy controls	Serum	n/a	qRT-PCR
^44^	China	Unclear	Normal samples	Whole blood	GEO dataset screening	qRT-PCR, 2^−ΔΔCT^ACTIN control
^45^	China	MRI or CT	Healthy controls, no history of stroke	Serum	n/a	U6 snRNAqRT-PCR, 2^−ΔCT^
^46^	China	Clinical diagnosisMRI or CT	Healthy volunteers	Plasma	n/a	U6 controlqRT-PCR, 2^−ΔΔCT^
^47^	China	Clinical diagnosisCT	Healthy volunteers	Plasma	n/a	cel-miR-39 controlqRT-PCR, 2^−ΔΔCT^
^48^	China	MRI or CT	Control no AIS	Serum		U6 controlqRT-PCR
^49^	China	Clinical diagnosisMRI or CT	Healthy volunteers	Blood	n/a	U6 controlqRT-PCR, 2^−ΔCt^
^50^	China	Unclear	Healthy controls	Serum	GEO Microarray	cel-miR-39 controlqRT-PCR, 2^−ΔΔCT^
^51^	China	Clinical diagnosisMRI or CT	Healthy participants	Blood	n/a	U6 controlqRT-PCR
^52^	China	Clinical diagnosisMRI	Healthy cases	Serum	n/a	U6 controlqRT-PCR
^53^	China	Clinical diagnosisMRI & MRA	Healthy individuals	Plasma	n/a	cel-miR-39 controlqRT-PCR, CT
^54^	China	Clinical diagnosis MRI or CT	Healthy controls	Serum	n/a	U6 controlqRT-PCR, 2^−ΔΔCt^
^55^	China	MRI or CT	Controls	Serum & serum derived EVs	n/a	U6 control qRT-PCR

CSF: cerebral spinal fluid, CT: computerised tomography, DWI: diffusion weight imaging, EVs: extracellular vesicles, MRI: magnetic resonance imaging, MRA: magnetic resonance angiography.

**Table 2.** Characteristics of patients with ischaemic stroke and healthy controls included.

	Ischaemic stroke							Healthy control						
Ref	n	Age	M%	HT%	D%	HL%	S%	n	Age	M%	HT%	D%	HL%	S%
^1^	44	63 ± 11	68	90	50	45	43	37	62 ± 10	65	51	11	37	30
^2^	50	64 ± 9	64	76	36	58	ns	33	63 ± 7	61	76	9	42	ns
^3^	128	68 ± 17	85	88	43	77	49	102	65 ± 16	73	67	37	33	52
^4^	6	49 ± 5	100	ns	0	ns	67	8	43 ± 7	ns	ns	0	ns	25
^5^	108	64 ± 9	69	53	32	60	ns	97	59 ± 6	46	47	59	4	ns
^6^	106	60 ± 9	45	76	32	49	25	110	58 ± 15	44	77	24	55	35
^7^	148	61 ± 9	62	80	2	52	50	148	59 ± 9	57	76	22	46	43
^8^	32	18–49	ns	ns	ns	ns	ns	14	18–49	ns	ns	ns	ns	ns
^9^	148	61 ± 9	72	60	24	ns	36	50	59 ± 10	72	40	10	ns	24
^10^	40	58 ± 2	65	ns	ns	ns	ns	45	57 ± 2	58	ns	ns	ns	ns
^11^	179	61–66	56	17	13	ns	18	50	64 ± 6	48	10	10	ns	20
^12^	85	61.2	51	51	28	ns	38	20	62	50	0	0	ns	25
^13^	83	66 ± 14	66	65	41	ns	ns	37	65 ± 11	41	60	22	ns	ns
^14^	65	64	61	66	28	28	ns	66	60	55	53	18	24	ns
^15^	24	62 ± 8	50	75	29	67	38	24	63 ± 8	50	88	33	67	20
^16^	106	62 ± 13	51	61	27	42	ns	106	60 ± 14	49	58	20	50	ns
^17^	20	54	33	ns	ns	ns	ns	20	55	31	ns	ns	ns	ns
^18^	8	61 ± 1	71	ns	ns	ns	ns	8	55 ± 2	72	ns	ns	ns	ns
^19^	44	63 ± 6	ns	ns	ns	ns	ns	22	64 ± 6	ns	ns	ns	ns	ns
^21^	93	72	51	74	34	18	30	23	ns	ns	ns	ns	ns	ns
^22^	117	68 ± 1	59	ns	ns	ns	ns	82	67 ± 1	58	ns	ns	ns	ns
^23^	30	62 ± 11	53	27	20	30	13	30	60 ± 8	46	60	0	13	40
^24^	146	67 ± 14	77	73	34	75	45	96	63 ± 15	66	86	34	35	43
^25^	31	66	68	84	29	68	36	11	ns	ns	ns	ns	ns	ns
^26^	14	60 ± 11	67	ns	0	ns	33	18	53 ± 11	64	ns	0	ns	57
^27^	72	72	57	32	24	26	22	51	71	58	0	0	0	17
^28^	20	35–67	65	ns	ns	ns	ns	20	33–71	60	ns	ns	ns	ns
^29^	88	62 ± 13	63	60	41	56	54	69	61 ± 13	64	62	39	53	52
^30^	56	53 ± 9	ns	0	100	ns	34	30	48 ± 8	ns	0	0	ns	27
^31^	10	73	50	ns	10	ns	ns	10	56.8	40	ns	0	ns	ns
^32^	21	66.6	57	ns	ns	ns	ns	21	66	61	ns	ns	ns	ns
^33^	54	68 ± 13	78	69	35	ns	31	51	65 ± 12	72	43	17	ns	29
^34^	19	18–49	53	63	53	ns	21	5	ns	60	ns	ns	ns	ns
^35^	13	67 ± 13	58	42	41	75	0	18	42 ± 16	67	0		0	0
^36^	7	68	70	67	24	42	27	4	63 ± 1	74	74	13	65	39
^37^	40	74	55	85	10	ns	50	40	69	40	65	5	ns	30
^20^	139	68	65	41	17	26	32	34	63	56	38	21	17	26
^38^	79	65 ± 10	78	77	39	61	ns	75	62 ± 6	67	36	5	37	ns
^39^	58	62	76	ns	ns	ns	ns	59	62	75	ns	ns	ns	ns
^40^	50	73 ± 12	48	24	12	ns	40	50	74 ± 12	52	32	14	ns	38
^41^	78	60 ± 10	70	65	19	24	ns	39	61 ± 5	71	39	21	77	ns
^42^	100	55 ± 6	65	57	23	ns	42	100	56 ± 7	71	47	18	ns	49
^43^	40	63	55	ns	ns	ns	ns	40	63	53	ns	ns	ns	ns
^44^	5	50-83	40	ns	ns	ns	ns	5	50-83	40	ns	ns	ns	ns
^45^	106	64 ± 11	52	60	52	ns	34	120	61 ± 19	48	36	32	ns	21
^46^	40	67 ± 11	58	ns	ns	ns	ns	39	65 ± 11	62	ns	ns	ns	ns
^47^	65	65 ± 6	61	64	24	26	ns	55	62 ± 7	51	52	18	23	ns
^48^	114	61 ± 11	68	80	32	21	43	58	56 ± 3	60	15	6	17	29
^49^	112	68	69	83	17	47	ns	60	67	51	n/a	n/a	n/a	ns
^50^	10	69 ± 10	ns	ns	ns	ns	ns	10	ns	ns	0	0	0	ns
^51^	10	55–65	100	ns	ns	ns	ns	12	55–65	100	ns	ns	ns	ns
^52^	75	54 ± 7	60	ns	ns	ns	ns	80	52 ± 8	62	ns	ns	ns	ns
^53^	68	64	66	54	25	11	26	21	58	52	0	0	0	0
^54^	108	66 ± 11	52	36	ns	ns	53	108	64 ± 13	57	24	ns	ns	45
^55^	10	76 ± 7	ns	ns	ns	ns	ns	10	ns	ns	ns	ns	ns	ns

M: male; HT: hypertension; D: diabetes mellitus; HL: hyperlipidaemia; S: smoking; NS: not shown.

#### Circulating microRNAs

**Figure 2.**
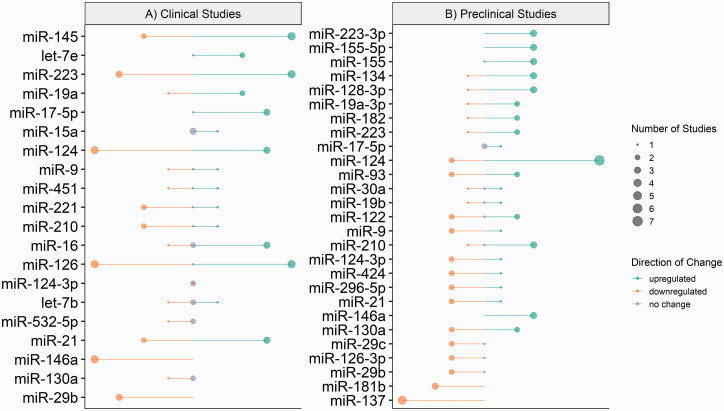
MicroRNA expression in included ischaemic clinical and preclinical studies. Diagrammatic representation of microRNA expression extracted from included ischaemic (a) clinical and (b) preclinical studies. Direction of change is indicated by colour; green – downregulation, orange – upregulated and lilac – no change. Circle size represents the number of included studies which reported expression. Constructed using Base R = R Core Team (2020) Vienna, Austria.

A total of 20 specific microRNAs were differentially expressed in more than two independent studies (Figure 2(a)). Of these, only two microRNAs were differentially expressed in the same direction across included literature; these were miR-146a and miR-29b.

Significant downregulation (0.34 to 0.88-fold) of miR-146a expression was reported in plasma, serum and whole blood samples from ischaemic stroke patients compared to healthy controls in four independent studies. Similarly, the expression of miR-29b was significantly downregulated (0.82 to 0.98-fold) in serum and blood samples from stroke patients compared to healthy controls in three independent studies.

Of the 20 microRNAs reported to have differential expression in clinical ischaemic stroke patient samples, within the current literature the expression of 15 microRNAs greatly varies in direction and magnitude when compared to healthy controls (Figure 2(a)).

### Ischaemic stroke – preclinical studies

#### Study design and methodology

The study design, experimental stroke model and methodology of included preclinical ischaemic stroke studies are summarised in Table 3.

**Table 3.** Study design of included preclinical ischaemic stroke studies.

Ref	Country	Animal species	Stroke induction	Sample type	Primary screening	microRNA quantification
^56^	Iran	Rat	MCA	Blood & brain tissue	n/a	U6 controlqRT-PCR, Ct
^57^	China	Rat	MCAO/R	Brain tissue	n/a	U6 controlqRT-PCR, 2^−ΔΔCT^
^58^	China	Mouse	MCAO	Brain tissue	n/a	U6 & GAPDH controlqRT-PCR, 2^−ΔΔCT^
^59^	China	Rat	PMCAO	Brain tissue	n/a	U6 controlqRT-PCR, 2^−ΔΔCq^
^60^	China	Mouse	MCAO	Brain tissue	n/a	β-actin & U6 controlqRT-PCR, 2^−ΔCT^
^61^	China	Mouse	MCAO	Brain tissue	n/a	β-actin & U6 controlqRT-PCR, 2^−ΔΔCT^
^62^	China	Rat	MCAO	Brain tissue	n/a	U6 controlqRT-PCR, 2^−ΔΔCt^
^63^	China	Mouse	MCAO	Brain tissue	n/a	U6 controlqRT-PCR
^64^	Brazil	Rat	MCAO	Brain tissue	n/a	U6 controlqRT-PCR
^65^	China	Rat	CCA ligation	Brain tissue	microArray	β-actin controlqRT-PCR, Ct
^66^	USA	Mouse	MCAO	Brain tissue	n/a	SnoRNA 202 controlqRT-PCR
^67^	China	Rat	MCAO	Brain tissue	microArray	β-actin & U6 controlqRT-PCR, 2^−ΔΔCT^
^68^	China	Rat	VO ischaemia	Brain tissue	n/a	β-actin controlqRT-PCR
^69^	USA	Rat	MCAO	Brain tissue	microArray	18s mRNA & GAPDH controlqRT-PCR, Ct
^70^	UK	Mouse	MCAO	Brain tissue	microArray	snRNA U6 control, RT-PCR
^71^	China	Rat	MCAO	Brain tissue	miRNA-seq	U6 controlqRT-PCR, 2^−ΔΔCq^
^72^	China	Mouse	MCAO	Brain tissue	n/a	GAPDH & U6 controlqRT-PCR, 2^−ΔΔCt^
^73^	China	Rat	MCAO	Brain tissue	n/a	U6 & GAPDH controlqRT-PCR, 2^−ΔΔCT^
^74^	China	Rat	MCA	Plasma & brain tissue	n/a	TNF-alpha control, qRT-PCR
^75^	Italy	Rat	tMCAO	Brain tissue	n/a	GAPDH controlqRT-PCR, ΔCT
^76^	Spain	Rat	MCAO	Brain tissue	n/a	snRNA U6 controlqRT-PCR, 2^−ΔΔCt^
^77^	China	Mouse	MCAO	Brain tissue	n/a	U6 controlqRT-PCR, 2^−ΔΔCt^
^78^	Russia	Rat	PI ischaemia	Brain tissue & leukocytes	RNA-seqTruSeq Small RNA	SNORD61 & SNORD72 controlqRT-PCR
^79^	Germany	Rat	tMCAO	Brain tissue	n/a	CycloA & GAPDH controlqRT-PCR, ΔΔCT
^80^	USA	Rat	MCAO	Brain tissue	microArray	snRNA U6 controlqRT-PCR
^81^	China	Mouse	MCAO	Brain tissue	n/a	U6 controlqRT-PCR, Cq
^82^	USA	Mouse	tMCAO	Brain tissue	RNA-seq	U6 & GAPDH controlqRT-PCR, 2^−ΔΔCT^
^83^	USA	Rat	MCAO	liver, muscle, brain tissue, plasma	n/a	qRT-PCR
^84^	China	Rat	MCAO	Blood & brain tissue	microArray	U6 controlqRT-PCR, 2^−ΔΔCt^
^85^	China	Rat	MCAO	Brain tissue	n/a	U6 controlqRT-PCR, 2^−ΔΔCt^
^86^	China	Rat	not shown	Brain tissue	n/a	U6 controlqRT-PCR, 2^−ΔΔCt^
^87^	China	Mouse	MCAO	Brain tissue	microArrayBeadChip	U6 controlqRT-PCR, ΔCt
^88^	USA	Mouse	MCAO embolus	SVZ cells, NP cells	n/a	U6 controlqRT-PCR, 2^−ΔΔCT^
^89^	Singapore	Rat	Embolus injection	Brain tissue	miRCURY LNA Array	qRT-PCR
^90^	China	Mouse	MCAO	Peri-infarct brain tissue	n/a	U6 controlqRT-PCR
^91^	China	Mouse	CIS suture	Brain tissue	n/a	U6 controlqRT-PCR, 2^−ΔCT^
^92^	USA	Rat	MCAO	Oligodendrocytes & NP cells	n/a	U6 controlqRT-PCR, 2^−ΔCT^
^93^	China	Mouse	IS model	Serum	n/a	U6 controlqRT-PCR, 2^−ΔCT^
^94^	China	Rat	I/R model	Brain tissue	n/a	U6 controlqRT-PCR, 2^−ΔΔCt^
^95^	China	Rat	HIBD	Brain tissue	n/a	U6 controlqRT-PCR, 2^−ΔΔCt^
^96^	China	Rat	MCAO	Brain tissue	n/a	qRT-PCR, 2^−ΔΔCt^
^97^	Italy	Mouse	MCAO	Brain tissue	microArray	qRT-PCR, ΔCTmiR
^98^	China	Mouse	MCAO	Ischaemic hemisphere	n/a	U6 controlqRT-PCR, 2^−ΔΔCT^
^99^	China	Rat	MCAO	Brain tissue	n/a	qRT-PCR
^100^	China	Mouse	MCAO	Brain tissue	n/a	qRT-PCR, 2^−ΔΔCt^
^101^	China	Mouse	MCAO	Brain tissue	MicroRNA chip	β-actin & U6 controlqRT-PCR, 2^−ΔΔCt^
^102^	China	Mouse	MCAO	Brain tissue	n/a	U6 controlqRT-PCR, 2^−ΔΔCt^
^103^	China	Mouse	MCAO	Brain tissue	RNAseq	U6 controlqRT-PCR, 2^−ΔΔCt^
^104^	China	Mouse	MCAO	Brain tissue	n/a	U6 controlqRT-PCR, ΔCT
^105^	China	Mouse	MCAO	Brain tissue	n/a	qRT-PCR
^106^	China	Mouse	Suture method	Brain tissue	n/a	snRNA U6 control, qRT-PCR
^107^	China	Rat	MCAO	Brain tissue	n/a	GAPDH & U6 controlqRT-PCR, 2^−ΔΔCt^
^108^	China	Rat	MCAO CIR	Brain tissue	n/a	U6 controlqRT-PCR
^109^	China	Rat	MCAO	Brain tissue	n/a	U6 controlqRT-PCR, 2^−ΔΔCt^
^110^	China	Rat	CCA ligation	Brain tissue	n/a	U6 controlqRT-PCR, 2^−ΔΔCt^
^111^	China	Rat	MCAO	Brain tissue	microArray	U6 controlqRT-PCR
^112^	China	Rat	Global cerebral ischaemia	Brain tissue	n/a	U6 controlqRT-PCR, 2^−ΔΔCt^
^113^	China	Mouse	MCAO	Brain tissue	n/a	miR-99 controlqRT-PCR, 2^−ΔΔCt^
^114^	Australia	Mouse	MCAO	Brain tissue	n/a	β-actin & U6 control, qRT-PCR
^115^	China	Mouse	TMCAO	Brain tissue	n/a	U6 & β-actin controlqRT-PCR, 2^−ΔΔCt^
^116^	China	Mouse	MCAO	Brain tissue	n/a	GAPDH controlqRT-PCR, 2^−ΔΔCq^
^117^	China	Mouse	MCAO	Brain tissue	microArray	U6 controlqRT-PCR, 2^−ΔΔCt^
^20^	UK	Rat	MCAO	Serum	n/a	cel-miR-39 controlqRT-PCR, ΔΔCt
^118^	China	Mouse	MCAO	Brain tissue	n/a	U6 controlqRT-PCR, 2^−ΔCT^
^119^	China	Mouse	MCAO	Brain tissue & blood	n/a	U6 controlqRT-PCR, 2^−ΔCt^
^40^	China	Mouse	MCAO	Brain tissue & blood	n/a	U6 controlqRT-PCR, 2^−ΔCt^
^120^	China	Mouse	MCAO	Brain tissue	n/a	U6 controlqRT-PCR
^121^	China	Rat	MCAO	Brain tissue	n/a	U6 controlqRT-PCR, 2^−ΔΔCT^
^122^	China	Rat	MCAO	Brain tissue	n/a	U6 controlqRT-PCR, 2^−ΔΔCt^
^123^	China	Rat	MCAO	Brain tissue	n/a	β-actin & U6 controlqRT-PCR, 2^−ΔΔCt^
^124^	China	Rat	CI - artery clamps	Brain tissue	n/a	β-actin controlqRT-PCR, 2^−ΔΔCt^
^125^	China	Rat	MCAO	Brain tissue	n/a	β-actin & U6 controlqRT-PCR, 2^−ΔΔCt^
^126^	China	Mouse	MCAO	Serum	n/a	U6 controlqRT-PCR
^127^	Japan	Rat	MCAO	Plasma	microArray ABI	qRT-PCR, Ct
^128^	China	Rat	MCAO	Brain tissue	n/a	qRT-PCR, 2^−ΔΔCt^
^129^	China	Rat	MCAO	Brain tissue	n/a	β-actin & U6 control
^130^	China	Rat	MCA	Brain tissue	n/a	qRT-PCR
^131^	China	Rat	HIBD	Brain tissue	n/a	U6 control, qRT-PCR
^132^	Taiwan	Rat	LMCA	Brain tissue	n/a	snRNA RNU6 controlqRT-PCR
^50^	China	Mouse	MCAO	Brain tissue & blood	n/a	U6 controlqRT-PCR, 2^−ΔCt^
^52^	China	Mouse	MCAO	Blood	n/a	U6 control, qRT-PCR
^133^	China	Mouse	Cerebral infarction	Brain tissue	n/a	U6 controlqRT-PCR, 2^−ΔΔCt^
^134^	China	Rat	MCAO	Brain tissue	n/a	U6 controlqRT-PCR, 2^−ΔΔCT^
^135^	China	Rat	MCAO	Brain tissue & blood	n/a	U6 control, qRT-PCR
^136^	China	Mouse	MCAO	Brain tissue & blood	n/a	qRT-PCR
^137^	China	Rat	MCAO	Brain tissue	n/a	U6 & GAPDH control, qRT-PCR
^138^	China	Rat	MCAO	Brain tissue & plasma	n/a	U6 controlqRT-PCR, Ct
^139^	China	Mouse	MCAO	Brain tissue	n/a	c. elegans & ACTB controlqRT-PCR, 2^−ΔΔCq^
^140^	China	Rat	MCAO	Brain tissue	n/a	U6 controlqRT-PCR, 2^−ΔΔCq^

CCA: common carotid artery; CI: cerebral ischaemia; CIS: cerebral ischaemic stroke; HIBD: hypoxic-ischaemic brain damage; LMCA: left middle cerebral artery occlusion; MCAO: middle cerebral artery occlusion; NP: neural progenitor; PMCAO: permanent middle cerebral artery occlusion; SVB: subventricular boundary; TMCAO: transient middle cerebral artery occlusion.

#### Circulating microRNAs

For preclinical models of ischaemic stroke, the extracted microRNA data included microRNA expression that was investigated in two or more independent studies is summarised in Figure 2(b). A total of 26 different microRNAs were assessed in more than two independent studies. Of these, 4 microRNAs were reported to be differentially expressed in the same direction across all included literature; these were miR-137, miR-146a, miR-181b and miR-223-3p.

In the current literature, 18 microRNAs reported altered expression in preclinical models of ischaemic stroke, with great variation in direction and magnitude when compared to healthy controls (Figure 2(b)).

### Haemorrhagic stroke – clinical studies

#### Study design and methodology

The study design and methodology of haemorrhagic stroke studies are summarised in Table 4. From the included studies, a summary of patient clinical data from haemorrhagic stroke cases and healthy controls are presented in Table 5.

**Table 4.** Study design of included clinical haemorrhagic stroke studies.

Ref	Country	Definition of haemorrhagic stroke	Definition of healthy control	Sample type	Primary screening	microRNA quantification
^141^	China	Clinical diagnosis CT or RI	Healthy controls	Blood	n/a	U6 control qRT-PCR, 2^−ΔCT^
^142^	China	Clinical diagnosis CT	Healthy controls	Blood	miRCURY LNA miRNA chips	miR-191-5p controlqRT-PCR, 2^−ΔCT^
^143^	China	Clinical diagnosis CT	Matched controls (sex, age and basic diseases)	Serum	n/a	U6 and GAPDH control qRT-PCR, 2^−ΔCT^
^144^	China	Clinical diagnosis MRI or CT	Healthy controls	Peripheral venous blood	n/a	RNU6B control qRT-PCR, 2^−ΔCT^
^145^	China	Clinical diagnosis MRI or CT	Patients without ICH	Plasma	n/a	miR-16 control qRT-PCR, 2^−ΔCT^

ICH: intracerebral haemorrhage; MRI: magnetic resonance imaging; CT: computerised tomography.

**Table 5.** Characteristics of patients with haemorrhagic stroke and healthy controls included.

	Haemorrhagic stroke							Healthy control						
Ref	n	Age	M%	HT%	D%	HL%	S%	n	Age	M%	HT%	D%	HL%	S%
^141^	65	62 ± 13	61	71	40	46	ns	69	61 ± 13	63	62	39	53	ns
^142^	33	57 ± 9	37	100	20	3 ± 1	28	15	58 ± 9	53	33	36	2 ± 0.5	36
^143^	30	60 ± 3	56	96	ns	ns	ns	30	61 ± 2	50	83	ns	ns	ns
^144^	80	64 ± 7	56	86	51	0	ns	30	ns	ns	ns	ns	ns	ns
^145^	106	56 ± 7	67	48	17	0	ns	50	ns	ns	ns	ns	ns	ns


M: male; HT: hypertension; D: diabetes mellitus; HL: hyperlipidaemia; S: smoking.

### Haemorrhagic stroke – preclinical studies

#### Study design and methodology

The study design, experimental model, and methodology of included preclinical haemorrhagic stroke studies are summarised in Table 6.

**Table 6.** Study design of included preclinical haemorrhagic stroke studies.

Ref	Country	Animal species	Method of stroke induction	Sample type	Primary screening	microRNA quantification
^146^	China	Rat	Collagenase induced ICH	Brain tissue	n/a	U6 control qRT-PCR, 2^−ΔCT^
^147^	China	Rat	Collagenase induced ICH	Brain tissue	n/a	U6 controlqRT-PCR
^148^	China	Rat	Collagenase induced ICH	Brain tissue	n/a	U6 controlqRT-PCR, 2^−ΔCT^
^149^	China	Rat	Collagenase induced ICH	Brain tissue	n/a	18S RNA control qRT-PCR, 2^−ΔCT^
^150^	China	Mice	Autologous blood injection	Brain tissue	n/a	qRT-PCR, 2^−ΔCt^

ICH: intracerebral haemorrhage.

#### Circulating microRNAs

**Figure 3.**
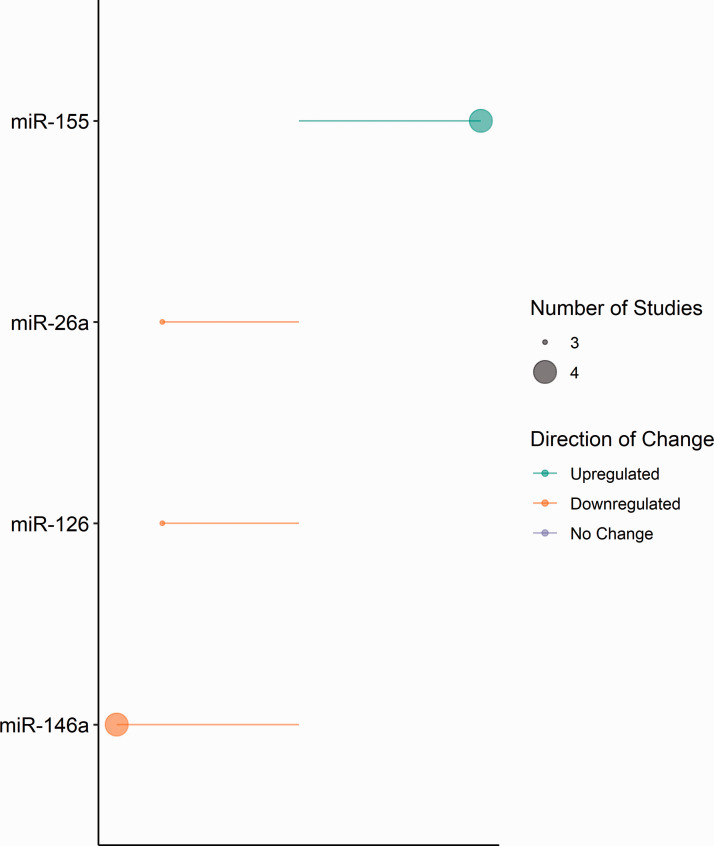
MicroRNA expression in included haemorrhagic clinical and preclinical studies. Diagrammatic representation of microRNA expression extracted from included haemorrhagic clinical and preclinical studies. Direction of change is indicated by colour; green – downregulation and orange – upregulated. Circle size represents the number of included studies reporting expression. Constructed using Base R = R Core Team (2020) Vienna, Austria.

For clinical and preclinical studies of haemorrhagic stroke, the extracted microRNA expression data that was investigated in more than two independent studies is summarised in Figure 3. A total of four microRNAs were reported as differentially expressed in more than two independent studies, namely miR-26a, miR-126, miR-146a and miR-155. Following haemorrhagic stroke, the expression of miR-26a (0.54 to 0.72-fold change), miR-126 (0.4 to 0.63-fold change), and miR-146a (0.33 to 0.81-fold change) were downregulated across clinical and preclinical studies.

### Combined ischaemic and haemorrhagic stroke subtypes

Data from microRNAs found to be altered across clinical and preclinical ischaemic and haemorrhagic stroke studies, which met the inclusion criteria and were investigated in two or more independent studies (Table 7). Only two microRNAs were reported to be differentially expressed in clinical and preclinical across both stroke subtypes: – miR-126 and miR-146a.

**Table 7.** MicroRNA expression in included both ischaemic and haemorrhagic studies.

MicroRNA	Stroke type	Relative Expression	Upregulated or downregulated	P Value	ROC analysis: AUC, P value
miR-126	Ischaemic^11,13,52,141,151–155^	1.03, 0.76, 0.934, 1.63, 11.72, 1.51, 0.43, 0.87, 0.28	No change, Downregulated, Upregulated	p = 0.216, p < 0.001, p = 0.148, p < 0.05, not determined	n/a, AUC = 0.654 (0.580–0.728), AUC = 0.665, AUC = 0.859 (0.792–0.925) p < 0.001, AUC = 0.92 (0.871–0.978), AUC = 0.8411, ns
	Haemorrhagic^141,146,156^	0.52, 0.40, 0.63	Downregulated	p = 0.026, p < 0.05, p < 0.01	n/a, AUC = 0.8411 (0.9085/0.7190), n/a
miR-146a	Ischaemic^4,19,52,157^	0.49, 0.88, 0.34, 0.42	Downregulated	p = 0.084, p < 0.001, p < 0.05	n/a, AUC = 0.91
	Haemorrhagic^143,147,148,156^	0.81, 0.33, 0.35, 0.64	Downregulated	p <0.05, p < 0.01, p <0.05, p < 0.01	n/a

Although consistently downregulated in haemorrhagic studies (0.4 to 0.63-fold change), across multiple ischaemic stroke studies, the direction of miR-126 expression was reported as either no change (1.03-fold change), upregulated (1.63 to 11.72-fold) or downregulated (0.28 to 0.93-fold change). In all current literature for both ischaemic and haemorrhagic stroke, miR-146a expression was consistently reported as downregulated in clinical and preclinical samples (0.33 to 0.88-fold change).

### Diagnostic potential of circulating microRNAs

To examine the diagnostic potential of differentially expressed microRNAs in ischaemic and haemorrhagic stroke patients in relation to healthy controls, 21 clinical studies described accuracy using receiver operator characteristic (ROC) analyses.

Of the 17 ischaemic stroke studies, the highest area under the curve (AUC) value was reported for miR-124 (AUC = 0.95). However, let-7b, let-7e, miR-124-3p, miR-126, miR-146a were each reported to have AUC values greater than 0.90.

Of the 4 haemorrhagic studies, miR-126 produced the highest AUC value (AUC = 0.84), while miR-26a produced the lowest (AUC = 0.69); no haemorrhagic studies reported an AUC value greater than 0.90.

### Prognostic potential of circulating microRNAs

Of the included literature, the short-term prognostic value of circulating microRNAs following ischaemic or haemorrhagic stroke was assessed in 6 studies: 4 with an ischaemic focus, 1 purely haemorrhagic and 1 which included both stroke subtypes.

In ischaemic stroke, the expression of miR-124 and patient prognosis was assessed through the Glasgow outcome scale (GOS); this indicated that downregulation of miR-124 in samples taken at 72 hrs post-stroke, correlated with unfavourable patient prognosis one-month post-stroke. The prognostic potential of miR-16, miR-29b and miR-223 were assessed at three months post-stroke; microRNA expression in samples taken at 72 hrs post-stroke, were compared to patients with good outcomes (modified Rankin Scale (mRS) ≤2) and those with poor outcomes (mRS > 2). Upregulated miR-29b expression was associated with good patient outcomes, while stroke patients with poor outcomes were found to have higher expression levels of miR-16 and miR-223, compared to patients with good outcomes.

In ischaemic and haemorrhagic stroke, the prognostic value of miR-126 was assessed at three months post-stroke. Patients with good prognosis had markedly higher levels of miR-126 expression at time of discharge, compared to those with poor prognosis. In haemorrhagic stroke, patients with downregulated miR-155 expression at time of admission suffered worse outcomes six-months post-stroke, in comparison with those with high miR-155 expression.

## Discussion

We identified that following ischaemic stroke, 20 microRNAs were consistently reported to be altered in clinical samples, 26 microRNAs were altered in preclinical ischaemic stroke models, while expression of four microRNAs were altered following clinical or preclinical haemorrhagic stroke.

In clinical ischaemic stroke studies, miR-146a and miR-29b were consistently downregulated. In preclinical ischaemic models miR-137, miR-146a, miR-181b and miR-223-3p were downregulated consistently; however, the magnitude of miR-223-3p expression varied substantially across studies. In haemorrhagic clinical and preclinical studies, microRNAs miR-26a, miR-126, and miR-146a were consistently downregulated, while miR-155 was consistently upregulated. Across ischaemic and haemorrhagic stroke studies, only miR-126 and miR-146a were reported to be differentially expressed in both stroke subtypes.

In the setting of stroke, recent preclinical literature states that miR-126 overexpression attenuates blood-brain barrier (BBB) disruption, promotes functional recovery, suppresses microglial activation, and improves neurogenesis following experimental stroke.^14,15^ Further, clinical ischaemic studies reported that circulating miR-126 correlates with low disease risk and reduced inflammatory levels in ischaemic patients.^16,17^ As for miR-146a, current preclinical literature indicates that circulating miR-146a can promote oligodendrogenesis^18^ and exosome-derived miR-146a can reduce microglial-induced neuroinflammation^19^ following stroke. Conversely, the inhibition of miR-146a led to exacerbated functional impairment, increased infarction volume, and increased BBB disruption.^20^ However, further studies are required to better understand the pathophysiological mechanisms and the molecular involvement of miR-126 and miR-146a following stroke; this will assist in developing and determining the therapeutic potential of microRNA modulation following stroke.

A previous review examined the association between acute ischaemic stroke and circulating microRNAs, focussing on clinical studies.^21^ The differential expression of 22 microRNAs was reported across 8 studies, with the expression of miR-106b-5p only being altered in more than one study. Importantly, the inclusion/exclusion criteria greatly differed to the current review, which will influence reporting of microRNA expression. Further, the previous systematic review did not include preclinical, or clinical haemorrhagic studies in their analysis.

Multiple factors could influence the differential expression of microRNAs between studies. Across clinical literature, the characteristics and risk factors of stroke patients varied, and the control groups ranged from those with similar risk factors to those with no risk factors (Tables 2 and 5). For 21 of the clinical ischaemic studies, comorbidities of stroke patients were unclear or unreported; therefore, it cannot be determined how these unreported factors may have impacted the results observed. In preclinical models, although rodents were used throughout current literature, the age, sex, breed, strain of the animals, and presence of comorbidity varied greatly across studies. Further, the definition of ‘sham animals’ also differed substantially across preclinical control groups, in addition to the group size and clinical characteristics of patients or experimental animals. Of note, the majority of patients included in ischaemic and haemorrhagic clinical literature were male (Tables 2 and 5); this sexual discrepancy was reflected dramatically in preclinical animals.

Further, in clinical ischaemic studies, the specific classification of ischaemic stroke may contribute to the variation of microRNA expression. Ischaemic stroke can be defined as large-artery atherosclerotic stroke, cardioembolic stroke, small vessel or unclassified, which arise through differing pathological mechanisms, which may affect the post-stroke expression of circulating microRNAs. Previous work from our group supports this, as patients with small vessel disease consistently displayed the highest expression of miR-family-17, while those with large artery, cardioembolic or unclassified stroke did not, when compared to non-stroke healthy controls.^22^

In addition, the method of microRNA isolation, detection and assessment differed between studies, which may further impact the expression of reported microRNAs and circulating RNAs. Clinical studies utilised different fractions of blood, while preclinical studies tended to detect expression in brain tissue. In this setting, the contamination of platelet microparticles has been a concern when isolating microRNAs from plasma or serum samples. Although our study did not discriminate between sample/tissue type, this may explain inconsistent results obtained across studies. A minority of preclinical studies analysed specific regions of the infarcted hemisphere, compared to corresponding material from sham animals. Although our specific criteria did not exclude studies based on collection time of the sample; it should be noted that the time of sample collection may affect the relative concentrations of reported microRNAs.

Of the included studies, 21 utilised primary assessment of microRNA expression via microarray chips and 2 studies profiled samples via RNAseq to identify microRNAs with altered expression then selected for further assessment via the validation stage. Other studies assessed microRNA expression based on previous research or literature-based searches. Across all studies, a range of qRT-PCR normalisation techniques were employed during microRNA quantification, which may have affected the normalisation of microRNA expression (Tables 1, 3, 4 and 6).

The specific diagnostic potential of the reported microRNAs is difficult to assess as only 21 of the included studies (17 ischaemic and 4 haemorrhagic studies) performed relevant analyses. Of these, six microRNAs reported an AUC value greater than 0.90 in ischaemic stroke; namely, let-7b, let-7e, miR-124, miR-124-3p, miR-126, and miR-146a. Previously, preclinical rodent studies identified the differential expression of miR-126-3p and miR-146a in ischaemic brain tissue; which would suggest that these microRNAs are representative of the neurological response to ischaemic stroke. In addition, the prognostic potential of reported microRNAs is difficult to assess, as only 6 studies (4 ischaemic, 1 haemorrhagic and 1 combined) performed short-term prognostic analysis of microRNA expression. In ischaemic stroke, the altered expression of two microRNAs were associated with good patient outcomes (miR-29b and miR-126), while three were associated with poor patient outcomes (miR-16, miR-124 and miR-223). In haemorrhagic stroke, one microRNA correlated with good outcomes (miR-126), and one was associated with worse patient outcomes (miR-155). However, prognostic assessments were only taken at one, three or six-months post-stroke, and compared to circulating microRNA levels from samples taken at admission, which ranged from 6 to 72 hrs post-symptom onset. Therefore, to assess the diagnostic and prognostic potential of microRNAs identified in this review as biomarkers of ischaemic and haemorrhagic stroke, further in-depth research is required.

There are several limitations in this systematic review. Primarily, microRNAs excluded due to not being reported in more than two previous studies are not deemed invalid biomarkers of ischaemic or haemorrhagic stroke. Similarly, microRNAs reported across more than two experimental studies, provide further evidence supporting their potential use as novel biomarkers in this setting; however, this does not validate their use clinically and further research is required. Of the included studies, 11–14% of studies were of high quality via our CAMARADES quality assessment. This strongly suggests that, at present, the diagnostic potential of microRNA in the setting of stroke is poorly investigated; therefore, there is a need for high quality research to address this issue to assess the true potential of microRNAs as a biomarker of stroke. Secondly, as previously discussed, substantial heterogeneity exists within the study design, methodologies, and results of the included studies. Due to the fold-change variation, and contradicting direction of microRNA expression observed post-stroke, it is difficult to truly determine the potential of microRNAs for future diagnostics. Thirdly, our inclusion criteria were not limited by patient age, sample type, sample collection time or stroke severity. We used this approach to widely assess the altered expression of circulating microRNAs in patients and animals after stroke; however, further analyses could subdivide expression based on these confounding factors, as the time of analysis, patient age, and degree of stroke may reflect the magnitude of altered microRNA expression post-stroke. Finally, the current review assessed circulating microRNAs, and did not discriminate between extracellular or intracellular microRNAs, or the cellular source of microRNAs; however, a more accurate representation of altered microRNA expression in disease may be derived from the cargo of extracellular vesicles (EVs). In the current review, five clinical studies determined the expression of EV-derived microRNAs. As EVs derive their cargo from the contents of their originating cell, they are an attractive source of biomarkers for a variety of diseases. Evidence indicates that circulating microRNAs are primarily transported by EVs.^23^ Recently, there has been a significant increase in knowledge of the role of EV-derived microRNAs in pathological processes, especially during cancer initiation and progression.^24–26^

## Conclusions

In conclusion, from the included literature, miR-146a and miR-126 were the only two microRNAs to have been reported as differentially expressed clinically and preclinically following ischaemic or haemorrhagic stroke. The literature suggests a differential signature of circulating microRNAs following ischaemic or haemorrhagic stroke; however, it is essential that further studies employing larger and more diverse populations, with consistent methodologies, are conducted to validate and determine the true clinical value of microRNAs as biomarkers of ischaemic and haemorrhagic stroke.

## Supplemental Material

sj-pdf-1-jcb-10.1177_0271678X221085090 - Supplemental material for Systematic review: Association between circulating microRNA expression & stroke Click here for additional data file. Supplemental material, sj-pdf-1-jcb-10.1177_0271678X221085090 for Systematic review: Association between circulating microRNA expression & stroke by Josie L Fullerton, Josephine M Thomas, Laura Gonzalez-Trueba, Cara Trivett, Josie C van Kralingen, Stuart M Allan, Terence J Quinn and Lorraine M Work in Journal of Cerebral Blood Flow & Metabolism
