# Supplementary material for: Systematic review: Association between circulating microRNA expression & stroke
Source: J Cereb Blood Flow Metab. 2022 Mar 3;42(6):935–51. doi: 10.1177/0271678X221085090 (PMC9121526; doi:10.1177/0271678X221085090)
Supplement: sj-pdf-1-jcb-10.1177_0271678X221085090 - Supplemental material for Systematic review: Association between circulating microRNA expression & stroke [file sj-pdf-1-jcb-10.1177_0271678X221085090.pdf]

Supplementary Information

Search strategy

For the selected databases, the following search string was developed, which was combined with two concepts and keyword terms: - concept 1 = “stroke”; (cerebrovascular disorders/basal ganglia cerebrovascular disease/ brain ischaemia, or carotid artery disease/ or intracranial arterial disease/ or intracranial embolism and thrombosis/ or intracranial haemorrhages/ or stroke/ or brain infarction/ or vertebral artery dissection), OR (stroke or cerebrovascular or brain vascular or cerebral vascular, or cva orapoplex), OR (brain or cerebral or vertebrobasilar or hemisphere or intracranial or intracerebral or infratentorial or supratentorial or MCA or anterior circulation or posterior circulation or basal ganglia) and (ischaemic, or infarct, or thrombosis, or embolism), OR (brain or cerebral or intracerebral or intracranial or parenchymal or intraventricular or infratentorial or supratentorial or basal ganglia) and (haemorrhage or hemorrhage or haematoma or hematoma or bleed), OR (stroke or poststroke or post*stroke or cerebrovascular or brain vascular or cerebral vascular or cva or apoplexy or ischaemic attack or ischemic attack or tia or neurological deficit or SAH or AVM). Concept 2 – “microRNA”; (microRNA/ micro RNA/ miR or miRNA or mir), OR non-coding, OR (primary microRNA, primary miRNA, or pre-miRNA, or pri-miRNA), OR (small temporal RNA or stRNA). Search results were concept 1 AND concept 2 combined and limited to publications after 1993 until present (October 2021, inclusively).

Inclusion and Exclusion Criteria

As prespecified in the protocol (http://syrf.org.uk/protocols/ - published 22.8.17).

Inclusion criteria: - any experiments profiling microRNA expression in humans or animals with stroke; in animal models and patients of all ages and both sexes; with appropriate use of control non-stroke patients/animals with comorbidities; microRNA expression, where quantitative measure of microRNA expression is reported, where microRNA expression outcome data has then been quantitatively measured by quantitative reverse transcription-polymerase chain reaction (qRT-PCR); studies published in peer reviewed scientific journals; in all languages were included. As long as microRNA data could be extracted, studies were still included if a subset of the included participants/experimental groups met the inclusion criteria. MicroRNA expression and any summary quantitative measure of microRNA expression, where an estimate of precision had been reported; microRNA expression data which has been quantitatively measured by some form of qRT-PCR – as opposed to non-validated array data.

Exclusion criteria: - non-stroke studies (such as traumatic brain injury or subarachnoid haemorrhage), intervention trials that may influence microRNA expression, reviews, protocol papers, editorials; studies which do not report quantitative measure of microRNA expression, and do not validate their data with qRT-PCR; studies which do not use appropriate non-stroke controls; publications published before 1993 and not published in peer reviewed journals.

Data extraction

Details of interest included: - journal title, year of publication, animal model (sex, species, strain), stroke model or stroke subtype, functional outcome measure(s) (primary and secondary), methods of microRNA quantification, and expression levels of circulating microRNAs.

CAMARADES checklist

Peer reviewed publication, use of control non-stroke patients or animals with comorbidities, samples size calculation, statement of compliance with ethical requirements, statement of possible conflicts of interest, pre-specified inclusion, and exclusion criteria, reporting of patient/animals excluded from analysis, source of study funding reported. Specifically, for non-human studies, the following evaluations were made: - control of temperature, randomised group allocation/blinded induction of stroke, blinded assessment of outcome, statement of compliance with animal welfare requirements, and monitoring of physiological variables throughout the study.

Reference list of included studies

1. Cai D, Wei D, Chen S, Chen X, Li S, Chen W, He W. MiR-145 protected the cell viability of human cerebral cortical neurons after oxygen-glucose deprivation by downregulating EPHA4. *Life Sciences*. 2019;231.
2. Y. C, Y. S, J. H, M. Q, Y. Z, J. G, Z. Z, J. L. Increased circulating exosomal miRNA-223 is associated with acute ischemic stroke. *Frontiers in Neurology* [Internet]. 2017;8:57. Available from: http://journal.frontiersin.org/article/10.3389/fneur.2017.00057/full
3. Chen Z, Wang K, Huang J, Zheng G, Lv Y, Luo N, Liang M, Huang L. Upregulated Serum MiR-146b Serves as a Biomarker for Acute Ischemic Stroke. *Cellular Physiology and Biochemistry*. 2018;45:397–405.
4. Duan X, Zhan Q, Song B, Zeng S, Zhou J, Long Y, Lu J, Li Z, Yuan M, Chen X, et al. Detection of platelet microRNA expression in patients with diabetes mellitus with or without ischemic stroke. *Journal of Diabetes and its Complications* [Internet]. 2014;28:705–710. Available from: http://dx.doi.org/10.1016/j.jdiacomp.2014.04.012
5. Fu C, Chen S, Cai N, Liu Z, Wang P, Zhao J. Potential Neuroprotective Effect of miR-451 Against Cerebral Ischemia/Reperfusion Injury in Stroke Patients and a Mouse Model. *World Neurosurgery* [Internet]. 2019;130:e54–e61. Available from: https://doi.org/10.1016/j.wneu.2019.05.194
6. F. J, Xing J. AO - Jin FO http://orcid. org/0000-0001-7740-9582. Circulating pro-angiogenic and anti-angiogenic microRNA expressions in patients with acute ischemic stroke and their association with disease severity. *Neurological Sciences* [Internet]. 2017;38:2015–2023. Available from: http://link.springer.de/link/service/journals/10072/index.htm

7. F. J, Xing J. AO - Jin FO http://orcid. org/0000-0001-7740-9582. Circulating miR-126 and miR-130a levels correlate with lower disease risk, disease severity, and reduced inflammatory cytokine levels in acute ischemic stroke patients. *Neurological Sciences* [Internet]. 2018;39:1757–1765. Available from: http://link.springer.de/link/service/journals/10072/index.htm

8. Gan CS, Wang CW, Tan KS. Circulatory microRNA-145 expression is increased in cerebral ischemia. *Genetics and molecular research : GMR*. 2012;11:147–152.

9. Gao J, Yang S, Wang K, Zhong Q, Ma A, Pan X. Plasma miR-126 and miR-143 as Potential Novel Biomarkers for Cerebral Atherosclerosis. *Journal of Stroke and Cerebrovascular Diseases* [Internet]. 2019;28:38–43. Available from: https://doi.org/10.1016/j.jstrokecerebrovasdis.2018.09.008

10. Gong Z, Zhao S, Zhang J, Xu X, Guan W, Jing L, Liu P, Lu J, Teng J, Peng T, et al. Initial research on the relationship between let-7 family members in the. serum and massive cerebral infarction *Journal of the Neurological Sciences* [Internet]. 2016;361:150–157. Available from: http://dx.doi.org/10.1016/j.jns.2015.12.047

11. Long G, Wang F, Li H, Yin Z, Sandip C, Lou Y, Wang Y, Chen C, Wang DW. Circulating miR-30a, miR-126 and let-7b as biomarker for ischemic stroke in humans. *BMC Neurology*. 2013;13.

12. Gui YX, Xu ZP, Jin T, Zhang LS, Chen LL, Hong B, Xie F, Lv W, Hu XY. Using Extracellular Circulating microRNAs to Classify the Etiological Subtypes of Ischemic Stroke. *Translational Stroke Research*. 2019;10:352–361.

13. Kim JM, Jung KH, Chu K, Lee ST, Ban J, Moon J, Kim M, Lee SK, Roh JK. Atherosclerosis-Related Circulating MicroRNAs as a Predictor of Stroke Recurrence. *Translational Stroke Research*. 2015;6:191–197.

14. Ji Q, Ji Y, Peng J, Zhou X, Chen X, Zhao H, Xu T, Chen L, Xu Y. Increased brain-specific MiR-9 and MiR-124 in the serum exosomes of acute ischemic stroke patients. *PLoS ONE*. 2016;11:1–14.

15. Jickling GC, Ander BP, Zhan X, Noblett D, Stamova B, Liu D. MicroRNA expression in peripheral blood cells following acute ischemic stroke and their predicted gene targets. *PLoS ONE*. 2014;9.

16. Jickling GC, Ander BP, Shroff N, Orantia M, Stamova B, Dykstra-Aiello C, Hull H, Zhan X, Liu D, Sharp FR. Leukocyte response is regulated by microRNA let7i in patients with acute ischemic stroke. *Neurology*. 2016;87:2198–2205.

17. Jin X, Wang H, Yin S, Zhang Y. MicroRNA-19a mediates neuroprotection through the PTEN/AKT pathway in SK-N-SH cells after oxygen-glucose deprivation/reoxygenation injury. *General physiology and biophysics* [Internet]. 2020;39:259—268. Available from: https://doi.org/10.4149/gpb_2020001

18. Kong Y, Li S, Cheng X, Ren H, Zhang B, Ma H, Li M, Zhang XA. Brain Ischemia Significantly Alters microRNA Expression in Human Peripheral Blood Natural Killer Cells. *Frontiers in Immunology*. 2020;11:1–8.

19. Kotb HG, Ibrahim AH, Mohamed EF, Ali OM, Hassanein N, Badawy D, Aly EA. The expression of microRNA 146a in patients with ischemic stroke: An observational study. *International Journal of General Medicine*. 2019;12:273–278.

20. Kralingen JC van, Mcfall A, Ord ENJ, Coyle TF, Bissett M, Mcclure JD, Mccabe C, Macrae IM, Dawson J, Work LM. Altered Extracellular Vesicle MicroRNA Expression in Ischemic Stroke and Small Vessel Disease. *Translational Stroke Research* [Internet]. 2018;Available from: http://dx.doi.org/10.1007/s12975-018-0682-3

21. Leung LY, Chan CPY, Leung YK, Jiang HL, Abrigo JM, Wang DF, Chung JSH, Rainer TH, Graham CA. Comparison of miR-124-3p and miR-16 for early diagnosis of hemorrhagic and ischemic stroke. *Clinica Chimica Acta* [Internet]. 2014;433:139–144. Available from: http://dx.doi.org/10.1016/j.cca.2014.03.007

22. Li P, Teng F, Gao F, Zhang M, Wu J, Zhang C. Identification of Circulating MicroRNAs as Potential Biomarkers for Detecting Acute Ischemic Stroke.

23. Li SH, Chen L, Pang XM, Su SY, Zhou X, Chen CY, Huang LG, Li JP, Liu JL. Decreased miR-146a expression in acute ischemic stroke directly targets the Fbxl10 mRNA and is involved in modulating apoptosis. *Neurochemistry International* [Internet]. 2017;107:156–167. Available from: http://dx.doi.org/10.1016/j.neuint.2017.01.011

24. Jia L, Hao F, Wang W, Qu Y. Circulating miR-145 is associated with plasma high-sensitivity C-reactive protein in acute ischemic stroke patients. *Cell Biochemistry and Function*. 2015;33:314–319.

25. Liu Y, Zhang J, Han R, Liu H, Sun D, Liu X. Downregulation of serum brain specific microRNA is associated with inflammation and infarct volume in acute ischemic stroke. *Journal of Clinical Neuroscience* [Internet]. 2015;22:291–295. Available from: http://dx.doi.org/10.1016/j.jocn.2014.05.042

26. Long Y, Zhan Q, Yuan M, Duan X, Zhou J, Lu J, Li Z, Yu F, Zhou X, Yang Q, et al. The Expression of microRNA-223 and FAM5C in Cerebral Infarction Patients with Diabetes Mellitus. *Cardiovascular Toxicology*. 2017;17:42–48.

27. Peng G, Yuan Y, Wu S, He F, Hu Y, Luo B. MicroRNA let-7e Is a Potential Circulating Biomarker of Acute Stage Ischemic Stroke. *Translational Stroke Research*. 2015;6:437–445.

28. Peng Z, Li M, Tan X, Xiang P, Wang H, Luo Y, Yang Y, Huang H, Chen Z, Xia H, et al. miR-211-5p alleviates focal cerebral ischemia-reperfusion injury in rats by down-regulating the expression of COX2. *Biochemical Pharmacology* [Internet]. 2020;177:113983. Available from: https://doi.org/10.1016/j.bcp.2020.113983

29. Qi RQ, Liu H, Liu C, Xu Y, Liu C. Expression and short-term prognostic value of miR-126 and miR-182 in patients with acute stroke. *Experimental and Therapeutic Medicine*. 2019;

30. Yang S, Zhao J, Chen Y, Lei M. Biomarkers Associated with Ischemic Stroke in Diabetes Mellitus Patients. *Cardiovascular Toxicology*. 2016;16:213–222.

31. Sørensen SS, Nygaard AB, Nielsen MY, Jensen K, Christensen T. miRNA Expression Profiles in Cerebrospinal Fluid and Blood of Patients with Acute Ischemic Stroke. *Translational Stroke Research*. 2014;5:711–718.

32. Sørensen SS, Nygaard AB, Carlsen AL, Heegaard NHH, Bak M, Christensen T. Elevation of brain-enriched miRNAs in cerebrospinal fluid of patients with acute ischemic stroke. *Biomarker Research*. 2017;5:1–10.

33. Sun M, Hou X, Ren G, Zhang Y, Cheng H. Dynamic changes in miR-124 levels in patients with acute cerebral infarction. *International Journal of Neuroscience* [Internet]. 2019;129:649–653. Available from: https://doi.org/10.1080/00207454.2018.1513931

34. Tan KS, Armugam A, Sepramaniam S, Lim KY, Setyowati KD, Wang CW, Jeyaseelan K. Expression profile of microRNAs in young stroke patients. *PLoS ONE*. 2009;4:1–9.

35. J.R. T, K.S. T, F.L. Y, A. A, C.W. W, K. J. MicroRNAs regulating cluster of differentiation 46 (CD46) in cardioembolic and non-cardioembolic stroke. *PLoS ONE* [Internet]. 2017;12:e0172131. Available from: http://journals.plos.org/plosone/article/file?id=10.1371/journal.pone.0172131&type=printable

36. Tian C, Li Z, Yang Z, Huang Q, Liu J, Hong B. Plasma MicroRNA-16 Is a Biomarker for Diagnosis, Stratification, and Prognosis of Hyperacute Cerebral Infarction. *PLoS ONE*. 2016;11:1–16.

37. Tiedt S, Prestel M, Malik R, Schieferdecker N, Duering M, Kautzky V, Stoycheva I, Böck J, Northoff BH, Klein M, et al. RNA-seq identifies circulating MIR-125a-5p, MIR-125b-5p, and MIR-143-3p as potential biomarkers for acute ischemic stroke. *Circulation Research*. 2017;121:970–980.

38. Wang Y, Zhang Y, Huang J, Chen X, Gu X, Wang Y, Zeng L. Increase of circulating miR-223 and insulin-like growth factor-1 is associated with the pathogenesis of acute ischemic stroke in patients. 2014;1–7.

39. Wang Y, Huang J, Ma Y, Tang G, Liu Y, Chen X, Zhang Z, Zeng L, Wang Y, Ouyang YB, et al. MicroRNA-29b is a therapeutic target in cerebral ischemia associated with aquaporin 4. *Journal of Cerebral Blood Flow and Metabolism* [Internet]. 2015;35:1977–1984. Available from: http://dx.doi.org/10.1038/jcbfm.2015.156

40. Wang L, Wang C, Xu L, Hua D. The expression research of miR-210 in the elderly patients with COPD combined with ischemic stroke. *European Review for Medical and Pharmacological Sciences*. 2016;20:4756–4760.

41. Wang Y, Ma Z, Kan P, Zhang B. The Diagnostic Value of Serum miRNA-221-3p, miRNA-382-5p, and miRNA-4271 in Ischemic Stroke. *Journal of Stroke and Cerebrovascular Diseases* [Internet]. 2017;26:1055–1060. Available from: http://dx.doi.org/10.1016/j.jstrokecerebrovasdis.2016.12.019

42. Wang X, Wang P, Yu L, He Y, Wang J. Serum miR-155 and its target gene hypoxiainducible factor 1α (HIF1A) are associated with acute ischemic stroke. *International Journal of Clinical and Experimental Medicine*. 2018;11:3740–3748.

43. Wang J, Huang Q, Ding J, Wang X. Elevated serum levels of brain-derived neurotrophic factor and miR-124 in acute ischemic stroke patients and the molecular mechanism. *3 Biotech* [Internet]. 2019;9:1–6. Available from: https://doi.org/10.1007/s13205-019-1914-2

44. He W, Chen S, Chen X, Li S, Chen W. Bioinformatic Analysis of Potential microRNAs in Ischemic Stroke. *Journal of Stroke and Cerebrovascular Diseases* [Internet]. 2016;25:1753–1759. Available from: http://dx.doi.org/10.1016/j.jstrokecerebrovasdis.2016.03.023

45. Wu J, Du K, Lu X. Elevated expressions of serum miR-15a, miR-16, and. 2015;8:21071–21079.

46. Yang Z-B, Li T-B, Zhang Z, Ren K-D, Zheng Z-F, Peng J, Luo X-J. The Diagnostic Value of Circulating Brain-specific MicroRNAs for Ischemic Stroke. *Internal Medicine* [Internet]. 2016;55:1279–1286. Available from: https://www.jstage.jst.go.jp/article/internalmedicine/55/10/55_55.5925/_article

47. Xiang W, Tian C, Lin J, Wu X, Pang G, Zhou L, Pan S, Deng Z. Plasma let-7i and miR-15a expression are associated with the effect of recombinant tissue plasminogen activator treatment in acute ischemic stroke patients. *Thrombosis Research* [Internet]. 2017;158:121–125. Available from: https://doi.org/10.1016/j.thromres.2017.09.004

48. Xue Y, Yin P, Li G, Zhong D. Genome-wide Integration Study of Circulating miRNAs and Peripheral Whole-Blood mRNAs of Male Acute Ischemic Stroke Patients. *Neuroscience* [Internet]. 2018;380:27–37. Available from: https://doi.org/10.1016/j.neuroscience.2018.04.001

49. Zeng L, Liu J, Wang Y, Wang L, Weng S, Tang Y, Zheng C, Cheng Q, Chen S, Yang G-Y. MicroRNA-210 as a novel blood biomarker in acute cerebral ischemia. 2011.

50. Zeng Y, Liu JX, Yan ZP, Yao XH, Liu XH. Potential microRNA biomarkers for acute ischemic stroke. *International Journal of Molecular Medicine*. 2015;36:1639–1647.

51. Zeng X, Wu J, Zhu Y, Yan G, Luo Z. Downregulation of microRNA 132-3p protects neural stem cells (NSC) against injury of cerebral ischemia (CI) via HO-1/Nrf2 signaling pathway. *International Journal of Clinical and Experimental Medicine*. 2018;11:132–3.

52. Zhu XY, Hou RY, Sun SC, Zhou XY. Serum miR-126 and miR-146a levels in patients with acute cerebral infarction and their relationship with severity of the disease. *Journal of Acute Disease* [Internet]. 2016;5:393–396. Available from: http://dx.doi.org/10.1016/j.joad.2016.08.005

53. Zhou J, Zhang J. Identification of miRNA-21 and miRNA-24 in plasma as potential early stage markers of acute cerebral infarction. *Molecular Medicine Reports*. 2014;10:971–976.

54. Zhou X, Qi L. miR-124 Is Downregulated in Serum of Acute Cerebral Infarct Patients and Shows Diagnostic and Prognostic Value. *Clinical and Applied Thrombosis/Hemostasis* [Internet]. 2021;27:107602962110354. Available from: http://journals.sagepub.com/doi/10.1177/10760296211035446

55. Qi Z, Zhao Y, Su Y, Cao B, Yang JJ, Xing Q. Serum Extracellular Vesicle–Derived miR-124-3p as a Diagnostic and Predictive Marker for Early-Stage Acute Ischemic Stroke. *Frontiers in Molecular Biosciences*. 2021;8.

56. Arab L, Fanni A, Nemati S, Arefian E, Ai J, Mokhtari T, Farahmandfar M, Aghdami N, Hassanzadeh G. Human embryonic derived neural progenitor cells improves neurological scores following brain ischemia/ reperfusion: Modulation of blood and brain tissue MicroRNA-210. *Journal of Contemporary Medical Sciences*. 2020;6:103–108.

57. Chai Z, Gong J, Zheng P, Zheng J. Inhibition of miR-19a-3p decreases cerebral ischemia/reperfusion injury by targeting IGFBP3 in vivo and in vitro. *Biological Research* [Internet]. 2020;53:1–11. Available from: https://doi.org/10.1186/s40659-020-00280-9

58. Chen F, Zhang L, Wang E, Zhang C, Li X. LncRNA GAS5 regulates ischemic stroke as a competing endogenous RNA for miR-137 to regulate the Notch1 signaling pathway. *Biochemical and Biophysical Research Communications* [Internet]. 2018;496:184–190. Available from: https://doi.org/10.1016/j.bbrc.2018.01.022

59. Chen Z, Yang J, Zhong J, Luo Y, Du W, Hu C, Xia H, Li Y, Zhang J, Li M, et al. MicroRNA-193b-3p alleviates focal cerebral ischemia and reperfusion-induced injury in rats by inhibiting 5-lipoxygenase expression. *Experimental Neurology* [Internet]. 2020;327:113223. Available from: https://doi.org/10.1016/j.expneurol.2020.113223

60. Chi W, Meng F, Li Y, Li P, Wang G, Cheng H, Han S, Li J. Impact of microRNA-134 on neural cell survival against ischemic injury in primary cultured neuronal cells and mouse brain with ischemic stroke by targeting HSPA12B. *Brain Research* [Internet]. 2014;1592:22–33. Available from: http://dx.doi.org/10.1016/j.brainres.2014.09.072

61. Chi W, Meng F, Li Y, Wang Q, Wang G, Han S, Wang P, Li J. Downregulation of miRNA-134 protects neural cells against ischemic injury in N2A cells and mouse brain with ischemic stroke by targeting HSPA12B. *Neuroscience*. 2014;277:111–122.

62. Chi L, Jiao D, Nan G, Yuan H, Shen J, Gao Y. miR-9-5p attenuates ischemic stroke through targeting ERMP1-mediated endoplasmic reticulum stress. *Acta Histochemica* [Internet]. 2019;121:151438. Available from: https://doi.org/10.1016/j.acthis.2019.08.005

63. B. C, Y. Z, H. Z, L. L, L. S. The role of microRNA-146a in regulating the expression of IRAK1 in cerebral ischemia-reperfusion injury. *Canadian Journal of Physiology and Pharmacology* [Internet]. 2018;96:611–617. Available from: http://www.nrc.ca/cgi-bin/cisti/journals/rp/rp_desy_e?cjpp

64. Cirino MLA, Porsani LB, Lizarte Neto FS, Tazima MFGS, Zimak RF, Carlotti CG, Colli BO, Tirapelli LF, Tirapelli DPC. Expression of miR-15b, miR-29b, miR-219 and miR-222 micrornas in rats with focal cerebral ischemia submitted to physical exercise. *Genetics and Molecular Research*. 2019;18.

65. Cui H, Yang L. Analysis of microRNA expression detected by microarray of the cerebral cortex after hypoxic-ischemic brain injury. *Journal of Craniofacial Surgery*. 2013;24:2147–2152.

66. Yin KJ, Deng Z, Huang H, Hamblin M, Xie C, Zhang J, Chen YE. miR-497 regulates neuronal death in mouse brain after transient focal cerebral ischemia. *Neurobiology of Disease*. 2010;38:17–26.

67. Deng B, Bai F, Zhou H, Zhou D, Ma Z, Xiong L, Wang Q. Electroacupuncture enhances rehabilitation through miR-181b targeting PirB after ischemic stroke. *Scientific Reports*. 2016;6:1–14.

68. Deng XH, Zhong Y, Gu LZ, Shen W, Guo J. MiR-21 involve in ERK-mediated upregulation of MMP9 in the rat hippocampus following cerebral ischemia. *Brain Research Bulletin* [Internet]. 2013;94:56–62. Available from: http://dx.doi.org/10.1016/j.brainresbull.2013.02.007

69. Dharap A, Bowen K, Place R, Li LC, Vemuganti R. Transient focal ischemia induces extensive temporal changes in rat cerebral MicroRNAome. *Journal of Cerebral Blood Flow and Metabolism*. 2009;29:675–687.

70. Dhiraj DK, Chrysanthou E, Mallucci GR, Bushell M. miRNAs-19b, -29b-2* and -339-5p show an early and sustained up-regulation in ischemic models of stroke. *PLoS ONE*. 2013;8.

71. Duan X, Gan J, Peng D, Bao Q, Xiao L, Wei L, Wu J. Identification and functional analysis of microRNAs in rats following focal cerebral ischemia injury. *Molecular Medicine Reports*. 2019;49:4175–4184.

72. Gao N, Tang H, Gao L, Tu GL, Luo H, Xia Y. LncRNA H19 Aggravates Cerebral Ischemia/Reperfusion Injury by Functioning as a ceRNA for miR-19a-3p to Target PTEN. *Neuroscience* [Internet]. 2020;437:117–129. Available from: https://doi.org/10.1016/j.neuroscience.2020.04.020

73. Ge XL, Wang JL, Liu X, Zhang J, Liu C, Guo L. Inhibition of miR-19a protects neurons against ischemic stroke through modulating glucose metabolism and neuronal apoptosis. *Cellular and Molecular Biology Letters*. 2019;24:1–11.

74. Geng W, Tang H, Luo S, Lv Y, Liang D, Kang X, Hong W. Exosomes from mirna-126-modified adscs promotes functional recovery after stroke in rats by improving neurogenesis and suppressing microglia activation. *American Journal of Translational Research*. 2019;11:780–792.

75. Greco R, Demartini C, Zanaboni AM, Blandini F, Amantea D, Tassorelli C. Endothelial nitric oxide synthase inhibition triggers inflammatory responses in the brain of male rats exposed to ischemia-reperfusion injury. *Journal of Neuroscience Research*. 2018;96:151–159.

76. Gubern C, Camõs S, Ballesteros I, Rodríguez R, Romera VG, Cañadas R, Lizasoain I, Moro MA, Serena J, Mallolas J, et al. MiRNA expression is modulated over time after focal ischaemia: Up-regulation of miR-347 promotes neuronal apoptosis. *FEBS Journal*. 2013;280:6233–6246.

77. Guo D, Ma J, Li T, Yan L. Up-regulation of miR-122 protects against neuronal cell death in ischemic stroke through the heat shock protein 70-dependent NF-κB pathway by targeting FOXO3. *Experimental Cell Research*. 2018;369:34–42.

78. Gusar VA, Timofeeva A v., Zhanin IS, Shram SI, Pinelis VG. Estimation of time-dependent microRNA expression patterns in brain tissue, leukocytes, and blood plasma of rats under photochemically induced focal cerebral ischemia. *Molecular Biology*. 2017;51:602–613.

79. Herzog R, Beyer C, Zendedel A, Lammerding L, Beyer C, Slowik A. Impact of 17beta-estradiol and progesterone on inflammatory and apoptotic microRNA expression after ischemia in a rat model. *Journal of Steroid Biochemistry and Molecular Biology*. 2017;167:126–134.

80. Hunsberger JG, Fessler EB, Wang Z, Elkahloun AG, Chuang DM. Post-insult valproic acid-regulated microRNAs: Potential targets for cerebral ischemia. *American Journal of Translational Research*. 2012;4:316–332.

81. Jiang D, Sun X, Wang S, Man H. Upregulation of miR-874-3p decreases cerebral ischemia/reperfusion injury by directly targeting BMF and BCL2L13. *Biomedicine and Pharmacotherapy* [Internet]. 2019;117:108941. Available from: https://doi.org/10.1016/j.biopha.2019.108941

82. Kobayashi M, Benakis C, Anderson C, Moore MJ, Poon C, Uekawa K, Dyke JP, Fak JJ, Mele A, Park CY, et al. AGO CLIP Reveals an Activated Network for Acute Regulation of Brain Glutamate Homeostasis in Ischemic Stroke. *Cell Reports* [Internet]. 2019;28:979-991.e6. Available from: https://doi.org/10.1016/j.celrep.2019.06.075

83. Laterza OF, Lim L, Garrett-Engele PW, Vlasakova K, Muniappa N, Tanaka WK, Johnson JM, Sina JF, Fare TL, Sistare FD, et al. Plasma microRNAs as sensitive and specific biomarkers of tissue injury. *Clinical Chemistry*. 2009;55:1977–1983.

84. Li S, Chen L, Zhou X, Li J, Liu J. MiRNA-223-3p and let-7b-3p as potential blood biomarkers associated with the ischemic penumbra in rats. *Acta Neurobiologiae Experimentalis*. 2019;79:205–216.

85. Li R, Li X, Wu H, Yang Z, Li FEI, Jianhong ZHU. Theaflavin attenuates cerebral ischemia/reperfusion injury by abolishing miRNA-128-3p-mediated Nrf2 inhibition and reducing oxidative stress. *Molecular Medicine Reports*. 2019;20:4893–9404.

86. Li J, Lv H, Che YQ. Long non-coding RNA Gas5 potentiates the effects of microRNA-21 downregulation in response to ischaemic brain injury. *Neuroscience* [Internet]. 2020;437:87–97. Available from: https://doi.org/10.1016/j.neuroscience.2020.01.014

87. Liu C, Peng Z, Zhang N, Yu L, Han S, Li D, Li J. Identification of differentially expressed microRNAs and their PKC-isoform specific gene network prediction during hypoxic pre-conditioning and focal cerebral ischemia of mice. *Journal of Neurochemistry*. 2012;120:830–841.

88. Liu XS, Chopp M, Wang XL, Zhang L, Hozeska-Solgot A, Tang T, Kassis H, Zhang RL, Chen C, Xu J, et al. MicroRNA-17-92 cluster mediates the proliferation and survival of neural progenitor cells after stroke. *Journal of Biological Chemistry*. 2013;288:12478–12488.

89. Liu FJ, Lim KY, Kaur P, Sepramaniam S, Armugam A, Wong PTH, Jeyaseelan K. MicroRNAs Involved in Regulating Spontaneous Recovery in Embolic Stroke Model. *PLoS ONE*. 2013;8.

90. Liu P, Zhao H, Wang R, Wang P, Tao Z, Gao L, Yan F, Liu X, Yu S, Ji X, et al. MicroRNA-424 protects against focal cerebral ischemia and reperfusion injury in mice by suppressing oxidative stress. *Stroke*. 2015;46:513–519.

91. Liu XL, Wang G, Song W, Yang WX, Hua J, Lyu L. microRNA-137 promotes endothelial progenitor cell proliferation and angiogenesis in cerebral ischemic stroke mice by targeting NR4A2 through the Notch pathway. *Journal of Cellular Physiology*. 2018;233:5255–5266.

92. X.S. L, M. C, W.L. P, X.L. W, B.Y. F, Y. Z, H. K, R.L. Z, X.M. Z. MicroRNA-146a Promotes Oligodendrogenesis in Stroke. *Molecular Neurobiology* [Internet]. 2017;54:227–237. Available from: http://www.springer.com/biomed/neuroscience/journal/12035

93. Liu NN, Dong ZL, Han LL. MicroRNA-410 inhibition of the TIMP2-dependent MAPK pathway confers neuroprotection against oxidative stress-induced apoptosis after ischemic stroke in mice. *Brain Research Bulletin* [Internet]. 2018;143:45–57. Available from: https://doi.org/10.1016/j.brainresbull.2018.09.009

94. Liu WG, Han LL, Xiang R. Protection of miR-19b in hypoxia/reoxygenation-induced injury by targeting PTEN. *Journal of Cellular Physiology*. 2019;234:16226–16237.

95. Liu J, Zhang S, Huang Y, Sun L. miR-21 protects neonatal rats from hypoxic-ischemic brain damage by targeting CCL3. *Apoptosis* [Internet]. 2020;25:275–289. Available from: https://doi.org/10.1007/s10495-020-01596-3

96. Lou YL, Guo F, Liu F, Gao FL, Zhang PQ, Niu X, Guo SC, Yin JH, Wang Y, Deng ZF. MiR-210 activates notch signaling pathway in angiogenesis induced by cerebral ischemia. *Molecular and Cellular Biochemistry*. 2012;370:45–51.

97. Lusardi TA, Murphy SJ, Phillips J, Chen Y, Davis CM, Young JM, Thompson SJ, Saugstad JA. MicroRNA responses to focal cerebral ischemia in male and female mouse brain. *Frontiers in Molecular Neuroscience*. 2014;7:1–9.

98. Mao G, Ren P, Wang G, Yan F, Zhang Y. MicroRNA-128-3p Protects Mouse Against Cerebral Ischemia Through Reducing p38α Mitogen-Activated Protein Kinase Activity. *Journal of Molecular Neuroscience*. 2017;61:152–158.

99. Meng YC, Ding ZY, Wang HQ, Ning LP, Wang C. Effect of microRNA-155 on angiogenesis after cerebral infarction of rats through AT1R/VEGFR2 pathway. *Asian Pacific Journal of Tropical Medicine* [Internet]. 2015;8:829–835. Available from: http://dx.doi.org/10.1016/j.apjtm.2015.09.009

100. Meng ZY, Kang HL, Duan W, Zheng J, Li QN, Zhou ZJ. MicroRNA-210 promotes accumulation of neural precursor cells around ischemic foci after cerebral ischemia by regulating the SOCS1-STAT3-VEGF-C pathway. *Journal of the American Heart Association*. 2018;7:1–18.

101. Miao W, Yan Y, Bao T hao, Jia W ji, Yang F, Wang Y, Zhu Y hong, Yin M, Han J hong. Ischemic postconditioning exerts neuroprotective effect through negatively regulating PI3K/Akt2 signaling pathway by microRNA-124. *Biomedicine and Pharmacotherapy* [Internet]. 2020;126:109786. Available from: https://doi.org/10.1016/j.biopha.2019.109786

102. Ouyang YB, Lu Y, Yue S, Xu LJ, Xiong XX, White RE, Sun X, Giffard RG. MiR-181 regulates GRP78 and influences outcome from cerebral ischemia in vitro and in vivo. *Neurobiology of Disease*. 2012;45:555–563.

103. Pan J, Qu M, Li Y, Wang L, Zhang L, Wang Y, Tang Y, Tian HL, Zhang Z, Yang GY. MicroRNA-126-3p/-5p Overexpression Attenuates Blood-Brain Barrier Disruption in a Mouse Model of Middle Cerebral Artery Occlusion. *Stroke*. 2020;619–627.

104. Peng Z, Li J, Li Y, Yang X, Feng S, Han S, Li J. Downregulation of miR-181b in mouse brain following ischemic stroke induces neuroprotection against ischemic injury through targeting heat shock protein A5 and ubiquitin carboxyl-terminal hydrolase isozyme L1. *Journal of Neuroscience Research*. 2013;91:1349–1362.

105. Qu M, Pan J, Wang L, Zhou P, Song Y, Wang S, Jiang L, Geng J, Zhang Z, Wang Y, et al. MicroRNA-126 Regulates Angiogenesis and Neurogenesis in a Mouse Model of Focal Cerebral Ischemia. *Molecular Therapy - Nucleic Acids* [Internet]. 2019;16:15–25. Available from: https://doi.org/10.1016/j.omtn.2019.02.002

106. Rong S, Zhang J. Modulatory role of microRNA-124 in targeting Ku70 during post-stroke neuronal apoptosis. *International Journal of Clinical and Experimental Pathology*. 2017;10:3697–3702.

107. Sha R, Han X, Zheng C, Peng J, Wang L, Chen L, Huang X. The effects of electroacupuncture in a rat model of cerebral ischemia-reperfusion injury following middle cerebral artery occlusion involves MicroRNA-223 and the PTEN signaling pathway. *Medical Science Monitor*. 2019;25:10077–10088.

108. Shi L, Liu H, Zhang M, Guo Y, Song C, Song D, Xia J, Xu Y. MiR-128-3p activates autophagy in rat brain cells after focal cerebral ischemia reperfusion through targeting Atg1. *International Journal of Clinical and Experimental Medicine*. 2016;9:2486–2492.

109. Shi L, Tian Z, Fu Q, Li H, Zhang L, Tian L, Mi W. miR-217-regulated MEF2D-HDAC5/ND6 signaling pathway participates in the oxidative stress and inflammatory response after cerebral ischemia. *Brain Research* [Internet]. 2020;1739:146835. Available from: https://doi.org/10.1016/j.brainres.2020.146835

110. Shi H, Xu Y, Cai W. Protective role of microRNA-454-3p in neonatal hypoxic-ischaemic encephalopathy by targeting ST18. *Biotechnology and Biotechnological Equipment* [Internet]. 2020;34:211–220. Available from: https://doi.org/10.1080/13102818.2020.1729861

111. Si W, Ye S, Ren Z, Liu X, Wu Z, Li Y, Zhou J, Zhang S, Li Y, Deng R, et al. MiR-335 promotes stress granule formation to inhibit apoptosis by targeting ROCK2 in acute ischemic stroke. *International Journal of Molecular Medicine*. 2019;43:1452–1466.

112. Sun JJ, Zhang XY, Qin XD, Zhang J, Wang MX, Yang JB. MiRNA-210 induces the apoptosis of neuronal cells of rats with cerebral ischemia through activating HIF-1α-VEGF pathway. *European Review for Medical and Pharmacological Sciences*. 2019;23:2548–2554.

113. Suofu Y, Wang X, He Y, Li F, Zhang Y, Carlisle DL, Friedlander RM. Mir-155 knockout protects against ischemia/reperfusion-induced brain injury and hemorrhagic transformation. *NeuroReport*. 2020;1:235–239.

114. Tabet F, Lee S, Zhu W, Levin MG, Toth CL, Cuesta Torres LF, Vinh A, Kim HA, Chu HX, Evans MA, et al. microRNA-367-3p regulation of GPRC5A is suppressed in ischemic stroke. *Journal of Cerebral Blood Flow and Metabolism*. 2020;40:1300–1315.

115. Tang C, Ou J, Kou L, Deng J, Luo S. Circ_016719 plays a critical role in neuron cell apoptosis induced by I/R via targeting miR-29c/Map2k6. *Molecular and Cellular Probes* [Internet]. 2020;49:101478. Available from: https://doi.org/10.1016/j.mcp.2019.101478

116. Tian F, Yuan C, Hu L, Shan S. MicroRNA-93 inhibits inflammatory responses and cell apoptosis after cerebral ischemia reperfusion by targeting interleukin-1 receptor-associated kinase 4. *Experimental and Therapeutic Medicine*. 2017;14:2903–2910.

117. Tian R, Wu B, Fu C, Guo K. miR-137 prevents inflammatory response, oxidative stress, neuronal injury and cognitive impairment via blockade of Src-mediated MAPK signaling pathway in ischemic stroke. *Aging*. 2020;12:10873–10895.

118. Wang P, Liang J, Li Y, Li J, Yang X, Zhang X, Han S, Li S, Li J. Down-regulation of miRNA-30a alleviates cerebral ischemic injury through enhancing Beclin 1-mediated autophagy. *Neurochemical Research*. 2014;39:1279–1291.

119. Wang Y, Zhang Y, Huang J, Chen X, Gu X, Wang Y, Zeng L, Yang G-Y. Increase of circulating miR-223 and insulin-like growth factor-1 is associated with the pathogenesis of acute ischemic stroke in patients [Internet]. 2014. Available from: http://www.biomedcentral.com/1471-2377/14/77

120. Wang P, Liang X, Lu Y, Zhao X, Liang J. MicroRNA-93 Downregulation Ameliorates Cerebral Ischemic Injury Through the Nrf2/HO-1 Defense Pathway. *Neurochemical Research* [Internet]. 2016;41:2627–2635. Available from: http://dx.doi.org/10.1007/s11064-016-1975-0

121. Wang J, Li D, Hou J, Lei H. Protective effects of geniposide and ginsenoside Rg1 combination treatment on rats following cerebral ischemia are mediated via microglial microRNA-155-5p inhibition. *Molecular Medicine Reports*. 2018;17:3186–3193.

122. Wang Y, Wang MD, Xia YP, Gao Y, Zhu YY, Chen SC, Mao L, He QW, Yue ZY, Hu B. MicroRNA-130a regulates cerebral ischemia-induced blood-brain barrier permeability by targeting Homeobox A5. *FASEB Journal*. 2018;32:935–944.

123. Wang M, Guo J, Dong LN, Wang JP. Cerebellar fastigial nucleus stimulation in a chronic unpredictable mild stress rat model reduces post-stroke depression by suppressing brain inflammation via the microRNA-29C/TNFRSF1A signaling pathway. *Medical Science Monitor*. 2019;25:5594–5605.

124. Wang Y, Gu J, Hu L, Kong L, Wang T, Di M, Li C, Gui S. miR-130a alleviates neuronal apoptosis and changes in expression of Bcl-2/Bax and caspase-3 in cerebral infarction rats through PTEN/PI3K/Akt signaling pathway. *Experimental and Therapeutic Medicine*. 2020;2119–2126.

125. Wang C, Wan H, Wang Q, Sun H, Sun Y, Wang K, Zhang C. Safflor Yellow B Attenuates Ischemic Brain Injury via Downregulation of Long Noncoding AK046177 and Inhibition of MicroRNA-134 Expression in Rats. *Oxidative Medicine and Cellular Longevity*. 2020;2020.

126. Wei N, Xiao L, Xue R, Zhang D, Zhou J, Ren H, Guo S, Xu J. MicroRNA-9 Mediates the Cell Apoptosis by Targeting Bcl2l11 in Ischemic Stroke. *Molecular Neurobiology* [Internet]. 2016;53:6809–6817. Available from: http://dx.doi.org/10.1007/s12035-015-9605-4

127. Weng H, Shen C, Hirokawa G, Ji X, Takahashi R, Shimada K, Kishimoto C, Iwai N. Plasma miR-124 as a biomarker for cerebral infarction. *Biomedical Research*. 2011;32:135–141.

128. Wu Y, Yao J, Feng K. miR-124-5p/NOX2 Axis Modulates the ROS Production and the Inflammatory Microenvironment to Protect Against the Cerebral I/R Injury. *Neurochemical Research* [Internet]. 2020;45:404–417. Available from: https://doi.org/10.1007/s11064-019-02931-0

129. Xu S ye, Jiang X li, Liu Q, Xu J, Huang J, Gan S wei, Lu W tian, Zhuo F, Yang M, Sun S quan. Role of rno-miR-124-3p in regulating MCT1 expression in rat brain after permanent focal cerebral ischemia. *Genes and Diseases*. 2019;6:398–406.

130. Xue XH, Wang HR, Su JL. Inhibition of MiR-122 decreases cerebral ischemia-reperfusion injury by upregulating dj-1-phosphatase and tensin homologue deleted on chromosome 10 (PTEN)/ Phosphonosinol-3 Kinase (PI3K)/AKT. *Medical Science Monitor*. 2020;26:1–13.

131. Yao K, Yang Q, Li Y, Lan T, Yu H, Yu Y. MicroRNA-9 mediated the protective effect of ferulic acid on hypoxic-ischemic brain damage in neonatal rats. *PLoS ONE* [Internet]. 2020;15:1–15. Available from: http://dx.doi.org/10.1371/journal.pone.0228825

132. Yuen CM, Yeh KH, Wallace CG, Chen KH, Lin HS, Sung PH, Chai HT, Chen YL, Sun CK, Chen CH, et al. EPO-cyclosporine combination therapy reduced brain infarct area in rat after acute ischemic stroke: Role of innate immune-inflammatory response, micro-RNAs and MAPK family signaling pathway. *American Journal of Translational Research*. 2017;9:1651–1666.

133. Zhang K, Zhu Y, Liu P, Ji R. MiR-124 inhibits neural apoptosis in ischemic stroke. *International Journal of Clinical and Experimental Pathology*. 2016;9:9924–9930.

134. W. Z. MicroRNA-124 expression in the brains of rats during early cerebral ischemia and reperfusion injury is associated with cell apoptosis involving STAT3. *Experimental and Therapeutic Medicine* [Internet]. 2019;17:2870–2876. Available from: http://www.spandidos-publications.com/etm/17/4/2870/download

135. Zhao LX, Li HZ, Guo RY, Ma T, Hou RY, Ma XW, Du YF. miR-137, a new target for post-stroke depression? *Neural Regeneration Research*. 2013;8:2441–2448.

136. Zhao H, Wang J, Gao L, Wang R, Liu X, Gao Z, Tao Z, Xu C, Song J, Ji X, et al. MiRNA-424 protects against permanent focal cerebral ischemia injury in mice involving suppressing microglia activation. *Stroke*. 2013;44:1706–1713.

137. Zheng T, Shi Y, Zhang J, Peng J, Zhang X, Chen K, Chen Y, Liu L. MiR-130a exerts neuroprotective effects against ischemic stroke through PTEN/PI3K/AKT pathway. *Biomedicine and Pharmacotherapy* [Internet]. 2019;117:109117. Available from: https://doi.org/10.1016/j.biopha.2019.109117

138. Zuo ML, Wang AP, Song GL, Yang ZB. miR-652 protects rats from cerebral ischemia/reperfusion oxidative stress injury by directly targeting NOX2. *Biomedicine and Pharmacotherapy* [Internet]. 2020;124:109860. Available from: https://doi.org/10.1016/j.biopha.2020.109860

139. Wang M, Liu X, Wu Y, Wang Y, Cui J, Sun J, Bai Y, Lang M-F. MicroRNA-122 protects against ischemic stroke by targeting Maf1. *Experimental and Therapeutic Medicine*. 2021;21.

140. Sun H, Li J-J, Feng Z-R, Liu H-Y, Meng A-G. MicroRNA-124 regulates cell pyroptosis during cerebral ischemia-reperfusion injury by regulating STAT3. *Experimental and Therapeutic Medicine*. 2020;20:1–1.

141. Qi RQ, Liu H, Liu C, Xu Y, Liu C. Expression and short-term prognostic value of miR-126 and miR-182 in patients with acute stroke. *Experimental and Therapeutic Medicine*. 2019;

142. Zhu Y, Wang J-L, He Z-Y, Jin F, Tang L. Association of Altered Serum MicroRNAs with Perihematomal Edema after Acute Intracerebral Hemorrhage. 2015;

143. Hu Y-L, Wang H, Huang Q, Wang G, Zhang H-B. MicroRNA-23a-3p promotes the perihematomal edema formation after intracerebral hemorrhage via ZO-1.

144. Maimaijiang A, Geng D, Yimamu Y, Lin L, Li D, Zhang Y. Prognostic value of serum miR-155 in intracerebral hemorrhage [Internet]. 2017. Available from: www.ijcep.com/

145. Gareev I, Yang G, Sun J, Beylerli O, Chen X, Zhang D, Zhao B, Zhang R, Sun Z, Yang Q, et al. Circulating MicroRNAs as Potential Noninvasive Biomarkers of Spontaneous Intracerebral Hemorrhage. *World Neurosurgery*. 2020;133:e369–e375.

146. Kong F, Zhou J, Zhou W, Guo Y, Li G, Yang L. Protective role of microRNA-126 in intracerebral hemorrhage. *Molecular Medicine Reports*. 2017;15:1419–1425.

147. Qu X, Wang N, Cheng W, Xue Y, Chen W, Qi M. MicroRNA-146a protects against intracerebral hemorrhage by inhibiting inflammation and oxidative stress. *Experimental and Therapeutic Medicine*. 2019;

148. Huan S, Jin J, Shi CX, Li T, Dai Z, Fu XJ. Overexpression of miR-146a inhibits the apoptosis of hippocampal neurons of rats with cerebral hemorrhage by regulating autophagy. *Human and Experimental Toxicology*. 2020;39:1178–1189.

149. Zhang W, Wang L, Wang R, Duan Z, Wang H. A blockade of microRNA-155 signal pathway has a beneficial effect on neural injury after intracerebral haemorrhage via reduction in neuroinflammation and oxidative stress. *Archives of Physiology and Biochemistry*. 2020;

150. Jin J, Zhou F, Zhu J, Zeng W, Liu Y. MiR-26a inhibits the inflammatory response of microglia by targeting HMGA2 in intracerebral hemorrhage. *Journal of International Medical Research* [Internet]. 48:1–11. Available from: https://us.sagepub.com/en-us/nam/open-access-at-sage

151. M. C, W. P, S. H. miR-126/VCAM-1 regulation by naringin suppresses cell growth of human non-small cell lung cancer. *Oncology Letters* [Internet]. 2018;16:4754–4760. Available from: http://www.spandidos-publications.com/ol/16/4/4754/download

152. Jin F, Xing J. Circulating pro-angiogenic and anti-angiogenic microRNA expressions in patients with acute ischemic stroke and their association with disease severity. *Neurological Sciences* [Internet]. 2017;38:2015–2023. Available from: https://doi.org/10.1007/s10072-017-3071-x

153. Jin F, Xing J. Circulating miR-126 and miR-130a levels correlate with lower disease risk, disease severity, and reduced inflammatory cytokine levels in acute ischemic stroke patients. *Neurological Sciences*. 2018;39:1757–1765.

154. J. G, S. Y, K. W, Q. Z, A. M. Plasma miR-126 and miR-143 as Potential Novel Biomarkers for Cerebral Atherosclerosis. *Journal of Stroke and Cerebrovascular Diseases* [Internet]. 2019;28:38–43. Available from: http://www.elsevier.com/inca/publications/store/6/2/3/3/6/9/index.htt

155. Tan KS, Armugam A, Sepramaniam S, Lim KY, Setyowati KD, Wang CW, Jeyaseelan K. Expression profile of microRNAs in young stroke patients. *PLoS ONE*. 2009;4.

156. Zhu Y, Wang JL, He ZY, Jin F, Tang L. Association of altered serum micro RNAs with perihematomal edema after acute intracerebral hemorrhage. *PLoS ONE*. 2015;10.

157. Li SH, Chen L, Pang XM, Su SY, Zhou X, Chen CY, Huang LG, Li JP, Liu JL. Decreased miR-146a expression in acute ischemic stroke directly targets the Fbxl10 mRNA and is involved in modulating apoptosis. *Neurochemistry International* [Internet]. 2017;107:156–167. Available from: http://dx.doi.org/10.1016/j.neuint.2017.01.011
